# The food-water quality nexus in periurban aquacultures downstream of Bangkok, Thailand

**DOI:** 10.1016/j.scitotenv.2019.133923

**Published:** 2019-12-10

**Authors:** Wojciech Mrozik, Soydoa Vinitnantharat, Thunchanok Thongsamer, Nipapun Pansuk, Pavinee Pattanachan, Parinda Thayanukul, Kishor Acharya, Marcos Quintela Baluja, Charles Hazlerigg, Aidan F. Robson, Russell J. Davenport, David Werner

**Affiliations:** a School of Engineering, Newcastle University, Newcastle upon Tyne, United Kingdom; b Pilot Plant Development and Training Institute, King Mongkut's University of Technology Thonburi, Bangkok 10140, Thailand; c School of Energy, Environment and Materials, King Mongkut's University of Technology Thonburi, Bangkok, 10140, Thailand; d Department of Environmental Engineering, Faculty of Engineering, King Mongkut's University of Technology Thonburi, Bangkok 10140, Thailand; e Enviresearch Ltd, Newcastle upon Tyne, United Kingdom

**Keywords:** Urbanization, Aquaculture, Eutrophication, Pesticides, Faecal source tracking, Antibiotic resistance genes

## Abstract

Peri-urban aquacultures produce nutritious food in proximity to markets, but poor surface water quality in rapidly expanding megacities threatens their success in emerging economies. Our study compared, for a wide range of parameters, water quality downstream of Bangkok with aquaculture regulations and standards. For parameters not meeting those requirements, we sought to establish whether aquaculture practice or external factors were responsible. We applied conventional and advanced methods, including micropollutant analysis, genetic markers, and 16S rRNA amplicon sequencing, to investigate three family-owned aquacultures spanning extensive, semi-intensive and intensive practices. Canals draining the city of Bangkok did not meet quality standards for water to be used in aquaculture, and were sources for faecal coliforms, *Bacteriodes*, *Prevotella*, Human *E. coli*, tetracycline resistance genes, and nitrogen into the aquaculture ponds. Because of these inputs, aquacultures suffered algae blooms, with and without fertilizer and feed addition to the ponds. The aquacultures were sources of salinity and the herbicide diuron into the canals. Diuron was detectable in shrimp, but not at a level of concern to human health. Given the extent and nature of pollution, peri-urban water policy should prioritize charging for urban wastewater treatment over water fees for small-scale agricultural users. The extensive aquaculture attenuated per year an estimated twenty population equivalents of nitrogen pollution and trillions of faecal coliform bacteria inputs from the canal. Extensive aquacultures could thus contribute to peri-urban blue-green infrastructures providing ecosystem services to the urban population such as flood risk management, food production and water pollution attenuation.

## Highlights

• Extensive, semi-intensive and intensive aquacultures near Bangkok were investigated.
• Aquaculture water quality was severely affected by urban pollution in the canals.
• Aquacultures released herbicides/algaecides into canals.
• Extensive aquacultures attenuated faecal pollution inputs from the canals.
• Extensive aquaculture could be integrated in blue-green infrastructures.

## Graphical abstract

**Figure:**
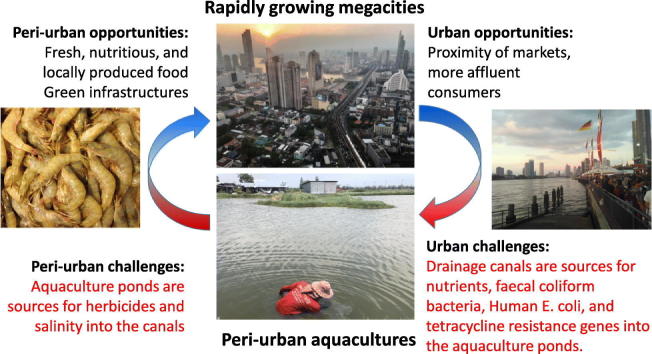

## 1 Introduction

The urbanization of emerging economies brings dramatic social, economic and environmental change that becomes particularly notable in the peri-urban regions around expanding megacities (Marshall and Dolley, 2019). The encroachment of urban into rural spaces creates both opportunities and conflicts (De Bon et al., 2010). An interesting case in point is peri-urban aquaculture. Aquaculture provides high quality, nutritious, protein-rich food to consumers, and improved earning opportunities for small-scale farmers in comparison with traditional crops like rice (Vagneron, 2007). Peri-urban aquaculture operations around Jakarta were observed to be adaptable and benefitted from urbanization due to their proximity to affluent markets (Pribadi and Pauleit, 2015), whereas paddy fields, food crops and livestock were mostly displaced by urbanization. A high level of female participation was noted in peri-urban oyster farming in Thailand which benefitted women with low levels of education and otherwise limited economic opportunities (Szuster et al., 2008). However, lack of adequate wastewater treatment, and poor surface water quality in the megacities of emerging economies pose significant threats to these peri-urban enterprises (Brooks and Conkle, 2019). For example, the peri-urban coastal areas in Thailand are considered unsuitable for the production of export quality shellfish owing to water quality concerns (Szuster et al., 2008).

Where aquacultures are relying on sewage influenced waters, the extent and implications of such de facto water reuse practices are poorly understood (Brooks and Conkle, 2019). According to the Pollution Control Department (PCD), Thailand's rapid urbanization is putting particular pressure on local surface water resources in the lower Chao Phraya River Basin, where the Bangkok Metropolis is located (PCD, 2016). Bangkok Metropolitan Administration (BMA) data for 2013 (Singkran, 2017) suggests that dissolved oxygen (DO), averaged over 286 observation stations across Bangkok, was only 2.1 mg/ L. The Thai national wastewater treatment capacity is only 33% of the total wastewater generated (PCD, 2016). In the Bangkok conurbation, domestic wastewater contains 51,897 tons of N/year, and only 45–53% of total domestic wastewater is treated (Buathong et al., 2013; Thitanuwat et al., 2016). BMA has invested 20 billion baht in building wastewater treatment facilities. In 2004, it first approved a policy to collect a treatment fee, but the policy failed to materialise. The most recent BMA proposal is for a tiered fee to be collected from the districts connected to wastewater treatment plants (Wancharoen, 2018). Fees would be calculated as 80% of the total amount of tap water used in each month in cubic metres, multiplied by two for households, four for office buildings and eight for large commercial users such as hotels and factories. The policy implies costs of 1.6, 3.2 and 6.4 baht per cubic meter, for each building classification respectively. BMA hopes to raise 800 million baht a year.

Thailand's Pollution Control Department has defined Coastal Water Quality Standards for water that is designated by fisheries laws to be used for aquacultural practices, and has also issued Coastal Sediment Quality Guidelines, as shown in Tables S1 and S2 in Supporting information. While aquacultures are dependent on good quality water resources, they may have their own negative impacts on surface water quality. Ferreira et al. (2015) estimated that the 2010 production of tilapia and shrimp in pond monocultures in Thailand corresponded to nitrogen emissions of 2,105,118 and 34,904 population equivalents, respectively. In 2004, Thailand's Ministry of Natural Resources and Environment defined Effluent Quality Standards for Coastal Aquaculture for ponds larger than 10 rai (Table S3 in Supporting information). A proposal is currently under review for a tiered Thai water user fee which would be free of charge for domestic and small-scale users, 0.5 baht per cubic meter abstracted for larger-scale use in agriculture, commerce, tourism, power generation and water supply, and 3 baht per cubic meter for water abstraction by big enterprises. Following concerns raised by academics and farmers, it was clarified that the proposed bill was not meant to tax small scale farmers irrigating their fields (Wipatayotin, 2018).

**Fig. 1:**
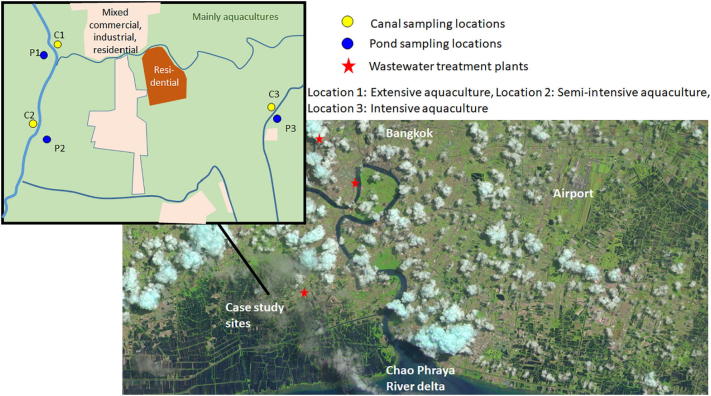
Approximate location and land use for the case study area.

Many stakeholders, including aquacultures, form a dense network of farming, industrial and housing activities in the peri-urban environment of Bangkok (Fig. 1). These activities depend on, and affect, local surface water quality. Insufficient understanding of water use and discharges by specific actors in the complex peri-urban environment are a major obstacle to sound planning and policy making (Vagneron, 2007). To address these knowledge gaps, we comprehensively assessed over a one year period water and sediment quality for three family-owned, peri-urban aquacultures which are situated along canals draining the city of Bangkok towards the Gulf of Thailand. Our study aim was to compare, for a wide range of parameters, the current water and sediment quality with the relevant guidelines and standards, and to establish for parameters, which were not compliant with the guidance, to what extent aquaculture management or external factors were responsible. From interviews with the farmers we sought evidence for water quality impacts on their enterprises, and a clearer understanding of aquaculture practices, including chemical, fertilizer and feed usages. The combined data enabled us to discuss the current food-water quality nexus for periurban aquacultures within the Bangkok Metropolitan Region, and implications for water policy making. The issues discussed in this paper are exemplary for other rapidly developing and urbanising regions in Southeast Asia and across the world (Brooks and Conkle, 2019).

## 2 Materials and methods

### 2.1 Case study site selection, interviews with farmers, and monitoring

Three case study aquaculture farms were selected in the Bang Khun Thian district of Bangkok and Phra Samut Chedi district of Samut Prakan province. They were located along canals which form part of the drainage system protecting the inner city of Bangkok from flooding (Fig. 1). These canals drain urban surface water towards the Gulf of Thailand. The exact locations of the farms cannot be revealed due to confidentiality agreements. They were small peri-urban enterprises owned by the farmers who participated voluntarily in the study, providing information about their businesses. The aquacultures spanned extensive, semi-intensive and intensive culturing practices. Extensive systems rely on photosynthetic production and have low stock densities. Semi-intensive systems provide extra nutrients and feeds, with medium stock densities, whereas intensive systems provide feeds and control the quality of their products by adding chemicals. Intensive systems normally need to provide oxygen to achieve high production with high stock densities.

### 2.2 Sampling schedule

Samples were taken on the same day from the ponds of each aquaculture (P), and adjacent canals (C), for the extensive (C1/P1), semi-intensive (C2/P2) and intensive (C3/P3) aquaculture (Fig. 1). Basic water quality analysis was done at King Mongkut's University of Technology Thonburi (KMUTT) once a month for 4 months each of the wet (June–September) and dry (February–May) seasons between 2017 and 18. In April 2017 and June 2018, filtered water samples were taken to Newcastle University (NU) for micronutrients and metals analysis. In March 2017, January, May, and June 2018, triplicate water samples from each location were solid-phase extracted at KMUTT and taken to NU for micropollutant analysis, together with air-dried sediment samples. In January, May, and June 2018 triplicate shrimp samples from aquaculture pond 2 were solid-phase extracted at KMUTT, and taken to NU for micropollutant analysis. In June 2018, duplicate water samples from each location were filtered at KMUTT, and DNA was extracted from the filters for molecular microbiology at NU. More details of the sampling are provided as Supporting information (Table S4).

### 2.3 Analytical methods

Comprehensive analytical method details are provided as Supporting information. Briefly, water samples were analysed for pH, dissolved oxygen (DO), electrical conductivity (EC) and temperature on site. Biochemical oxygen demand (BOD), chemical oxygen demand (COD), nitrite (NO_2_^−^-N), nitrate (NO_3_^−^-N), ammonia (NH_3_-N), ortho-phosphate phosphorus (Ortho-P), total phosphorus (TP), suspended solids (SS), fat, oil and grease (FOG), and faecal coliform bacteria were determined at KMUTT following Standard Methods for the Examination of Water and Wastewater (APHA, 2015). Metals in water samples were analysed at NU using a Varian Vista-MPX ICP-OES or Agilent 770 Series ICP-MS, as appropriate for the metal concentration. Metals in sediment were analysed commercially by Derwentside Environmental Testing Services Ltd., Consett, UK using accredited MCERTS methods. For molecular microbiology, water samples were first filtered, and total DNA was then extracted from bacterial biomass retained by the membrane. Total extracted DNA was sent to Department of Applied Biology, Cellular and Molecular Sciences, Northumbria University, UK for 16S rRNA amplicon sequencing. The V4 region of the 16S rRNA gene was targeted by using a previously published primer (Caporaso et al., 2010). The DNA were sequenced on the MiSeq Illumina Sequencing Platform using a previously published protocol (Kozich et al., 2013). In addition, real time polymerase chain reaction (PCR) assays were performed to quantify biomarkers for human-specific *E. coli* strains and tetracycline resistance genes on a BioRad CFX C1000 system (BioRad, Hercules, CA USA). The primers used are summarized in Table S5 in Supporting information. Analysis of antibiotics, pesticides and herbicides in water, sediments and shrimp was performed at NU with an UPLC-MS/MS system (Waters, Elstree, UK). Method parameters and validation data are summarized in Tables S6 and S7 in Supporting information. US EPA polycyclic aromatic hydrocarbons (PAHs) in sediment were analysed at NU by GC–MS (Agilent, Palo Alto, USA).

### 2.4 Risk assessment and statistical methods

A risk-based assessment for surface water in the form of a Toxicity Exposure Ratio (TER) was conducted for organic micropollutants. Combination toxicity was also assessed using a simple, additive model. Details of the risk assessment methodology are provided in Supporting information. Two-tailed, unpaired, heteroscedastic *t*-tests were used to assess differences between pond and canal water samples. Two-tailed one sample *t*-tests were used to test against the null hypothesis that net pollutant fluxes between ponds and canals were zero. All calculations and statistical tests were performed using Excel.

## 3 Results and discussion

### 3.1 Aquaculture practices

The aquaculture characteristics and management activities were gathered from interviews with the farmers (Table 1, Table 2). The extensive shrimp farmer did not add nutrients (i.e. fertilizer or feed) to the pond. For pond preparation he used saponin against invasive cichlids and lime to add alkalinity and kill germs. During culture he used dolomite, “effective microorganisms” (according to the label *Bacillus subtilis*, *Bacillus megaterium*, *Bacillus licheniformis*), and glyphosate to control weeds around the pond and excessive algae in the pond after drainage. The semi-intensive mixed shrimp/tilapia farmer used fertilizer during pond preparation to stimulate photosynthetic production, and regularly fed the fish. In addition, saponin was used to kill invasive cichlids, and smectite for pond preparation to control pH and reduce ammonia and hydrogen sulphide and to provide a source of silicate for diatoms and shrimp. Herbicides diuron and 2,4-dichlorophenoxyacetic acid (2,4-D) were used for the control of weeds and excessive algae blooms. The intensive seabass farm added fish feed twice a day. The owner used lime, the disinfectant chlorine, “effective microorganisms” and the insecticide dipterex for pond preparation, lime and zeolite during culture and diuron for weed/excessive algae growth control.

**Table 1:** Case study aquaculture farm characteristics.

	Farm 1	Farm 2	Farm 3
Aquaculture practice	Extensive	Semi-intensive	Intensive
Stock	White or Vannamei shrimp, black tiger shrimp	White or Vannamei shrimp and Tilapia, black tiger shrimp and Tilapia	Seabass
Pond area (m^2^)	Culture pond: 22400	Culture pond: 13981 Hatchery pond: 144 Storage pond: 1301	Culture pond: 3840 Hatchery ponds: 1200
Average depth (m)	0.7	0.7	1.8
Pond filling from canal	Culture pond: 100% of pond volume by natural gradient once a year	Culture pond: No Hatchery pond: 90% of pond volume by pump five times a year	Culture pond: 100% of pond volume by pump twice a year Hatchery pond: 100% of pond volume by pump once a year
Water intake from canal during culture	No	About 10% of pond volume, 4 times a month	10 months per year, 20% of pond volume by pump twice a month
Water discharge from pond into canal during culture	No	About 10% of pond volume, 4 times a month	10 months per year, 20% of pond volume by pump twice a month
Emptying the pond into the canal	90% of pond volume by pump once a year	No	90% of pond volume by pump twice a year
Sediment dredging	Every 1–1.5 years, onto earth dyke	Every 2–3 years, onto earth dyke	Every 2–3 years
Aeration	No	Hatchery only	Culture & Hatchery, 20 h per day
Feeding	No	Twice a day	Twice a day
Chemical usage	Pond preparation: Saponin, Lime Culture: Dolomite, Effective Microorganisms Herbicides: Glyphosate	Pond preparation: Saponin, Smectite, D Max (fertilizer) Culture: Fish feed Herbicides: Diuron, 2,4-D	Pond preparation: Lime, Chlorine, Effective Microorganisms, Dipterex Culture: Fish feed, Lime, Zeolite, Effective Microorganisms Herbicides: Diuron

**Table 2:** Case study aquaculture farm activities during the case study period.

	Farm 1	Farm 2	Farm 3
	Extensive	Semi-intensive	Intensive
Dec	White shrimp in pond		
Jan 2017		Add stock white shrimp ~200,000	Add stock seabass ~15,000
Feb		Add stock tilapia ~5000	
March	Algae bloom		
April	Pond drainage	Add stock white shrimp ~200,000	
May	Pond preparation/filling		Algae bloom, fish die
June	Change stocking from white to black tiger shrimp due to poor survival of the former and higher price of the later, stocking rate ~100,000		Harvest seabass ~4000 (economic loss)
July	Add stock black tiger shrimp ~50,000	Add stock white shrimp ~200,000	Pond preparation
Aug	Add stock black tiger shrimp ~50,000		Add stock seabass ~15,000
Sept	Add stock black tiger shrimp ~50,000	Add stock white shrimp ~200,000	Change fish feed due to prior algae bloom/feed left in pond
Oct	Add stock black tiger shrimp ~50,000	Harvest tilapia, add stock tilapia ~5000	
Nov	Add stock black tiger shrimp ~50,000		
Dec	Stopped all activities in pond due to poor harvest and shrimp being too small for sale.	Add stock white shrimp ~200,000	Low temperature, fish feed left in pond
Jan 2018		Add stock black tiger shrimp due to higher price of the later, ~200,000	Harvest seabass ~12,000
Feb			Pond preparation
March		Add stock black tiger shrimp ~200,000	Add stock seabass ~15,000
May		Add stock black tiger shrimp ~200,000	
July		Harvest tilapia	

All aquacultures exchanged water with the associated canals, but with different exchange patterns (Table 1). The extensive aquaculture filled the pond with canal water, and emptied pond water into the canal typically once a year, but did not exchange water during culture. According to the farmer, the lack of regular water exchange was because of poor canal water quality with odorous contaminants. The semi-intensive aquaculture did not fill or empty the culture pond during the monitoring period, but regularly exchanged water with the canal. The intensive aquaculture had the most extensive water exchange, filling and emptying the culture pond twice a year, and regularly exchanging water with the canal during culture.

### 3.2 Water quality problems reported by the farmers

Interviews with the farmers recorded several water pollution related problems. The extensive and intensive aquacultures 1 and 3 both reported algae blooms during the observation period, in March 2017 and May 2017, respectively (Table 2). In the intensive seabass culture, this algae bloom led to the death of fish and a low harvest in June, causing an economic loss for that culture cycle. At the extensive aquaculture, the owner stopped all aquaculture activities in December 2017, because few shrimp could be trapped, and those harvested were too small for sale. The farmer attributed these difficulties to poor water quality and invasive fish species of the Cichlidae family, which eat shrimp. The non-native Mayan cichlid fish (*Cichlasoma urophthalmus*) began appearing in the catches of Thai fishermen in 2005, and has since established itself in the lower Chao Phraya River delta region (Nico et al., 2007). The suspected source for these invasive species is the aquarium ornamental fish trade. The interviews with the farmers revealed that aquacultures were affected by poor water quality regardless of their practices. Both, the extensive and intensive aquacultures, suffered algae blooms, even though the former practice didn't add nutrients to the pond.

### 3.3 Peri-urban water quality in comparison with regulatory guidelines and standards for coastal aquaculture

**Fig. 2:**
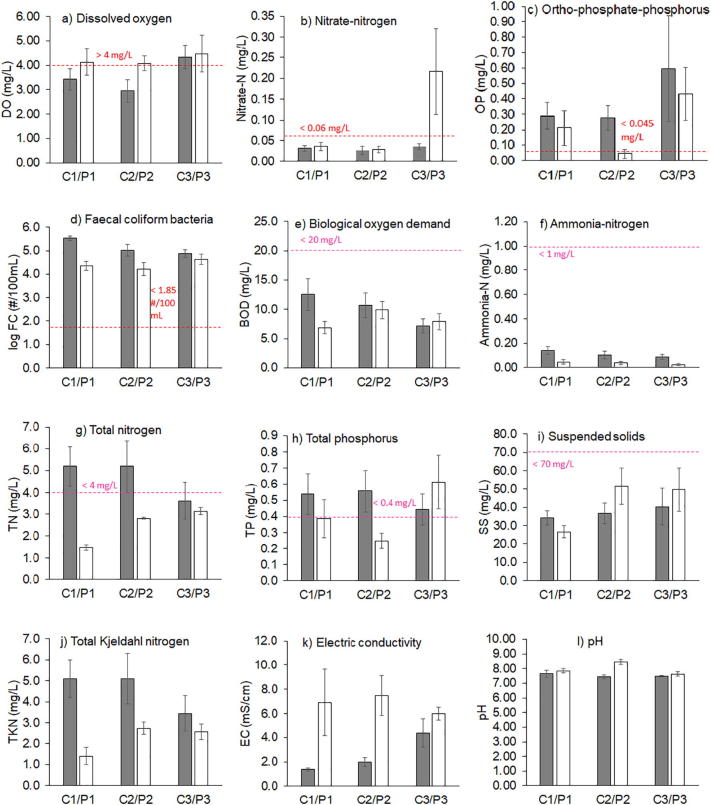
General water quality indicators in canals (C, grey bars) and ponds (P, white bars) for the extensive (C1/P1), semi-intensive (C2/P2) and intensive (C3/P3) aquaculture. Error bars indicate the standard errors of the mean (*n* = 6–8 sampling events). Coastal water quality standards for water to be used in aquaculture are indicated as red lines. Aquaculture effluent quality standards are indicated as pink lines. More detailed and additional data is available in Supporting information. (For interpretation of the references to colour in this figure legend, the reader is referred to the web version of this article.)

Classic water quality parameters measured in the canals and aquaculture ponds are illustrated in Fig. 2, and compiled in Table S8 in Supporting information. Canal water samples did not meet the requirements set out in the Thai Coastal Water Quality Standard for water to be used for aquaculture practices (class 3, Table S1 in Supporting information): 67% of samples had less than the required ≥4 mg/L dissolved oxygen, 63% exceeded 45 μg/L orthophosphate‑phosphorus, 17% exceeded ≤0.06 mg/L nitrate‑nitrogen, and 100% exceeded ≤70/100 mL faecal coliform bacteria. Dissolved micronutrient and metal concentrations (Table S9 in Supporting information) mostly met requirements, except for ≤100 μg/L manganese, which was exceeded in 83% of the samples. The measured water quality thus confirmed the perception by the farmers that canal water quality was very poor and did not meet their requirements.

On the other hand, water quality in the aquaculture ponds mostly complied with requirements set out in the Effluent Quality Standards for Coastal Aquaculture (Table S3 in Supporting information). The biological oxygen demand in ponds was <20 mg/L, but this level was occasionally exceeded in the canals (i.e. receiving water body, 13% of samples). Pond water concentrations of ammonia nitrogen and total nitrogen were generally in compliance, except for one sample from the intensive aquaculture pond 3, which slightly exceeded the total nitrogen standard in January 2018. In the canals, total nitrogen concentrations frequently exceeded 4.0 mg/L (57% of the samples), and reached concentrations as high as 11.42 mg/L. Total phosphorus in ponds sometimes exceeded 0.4 mg/L (33% of samples, mainly from the intensive aquaculture 3). However, in the canals, total phosphorus concentrations even more frequently exceeded this concentration level (58% of the samples). The pond suspended solids concentrations sometimes exceeded 70 mg/L (21% of the samples).

### 3.4 Seasonal trends

When comparing water samples from the dry and rainy season, the mean concentration values for faecal coliform bacteria were overall significantly higher in the dry season, whereas ortho-phosphate‑phosphorus were significantly higher in the rainy season (*t*-tests, *p* < 0.05). Rainfall could have diluted faecal coliforms from domestic wastewater discharges, whereas more run-off from agricultural land may have contributed to higher ortho-phosphate‑phosphorus concentration during the rainy season. These patterns thus suggested distinct sources for faecal coliform bacteria and ortho-phosphate pollution.

### 3.5 Pollutant fluxes between aquaculture ponds and canals

When comparing canal water quality with pond water quality, the mean concentration values for the parameters total nitrogen, total Kjedahl nitrogen, ammonia nitrogen, and faecal coliform bacteria were all significantly lower in ponds as compared to canals (*t*-tests, all *p* < 0.01). This implies that canals were net sources of nitrogen eutrophication and faecal pollution into the ponds, since volumes of water exchanged between ponds and canals were roughly balanced (Table 1). Note that in Bangkok precipitation and evaporation from aquaculture ponds are approximately equal over a calendar year (Yoo and Boyd, 2012). Mean values of electric conductivity and pH were significantly higher in ponds as compared to the respective canals (*t*-tests, all *p* < 0.01). Hence, the ponds were net sources for salinity into the canals. The electric conductivity of canal and pond water in the case study area was affected by tidal seawater intrusion. Farmers would have filled their ponds at high tide, when water would be more saline, using gravity flow. Addition of minerals like lime may have further enhanced salinity in the ponds. For the other basic water quality parameters, including chemical oxygen demand, fat oil and grease, and temperature (not shown in Fig. 1, but included in Table S8 in Supporting information), there were no statistically significant differences between mean values in ponds versus canals.

**Fig. 3:**
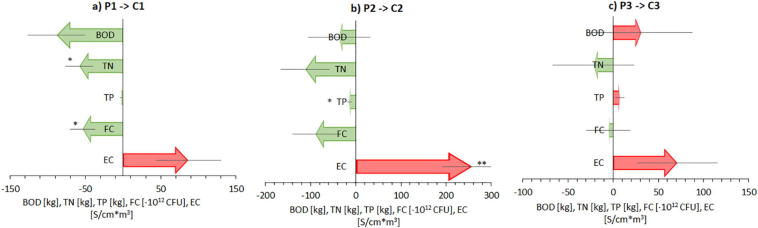
Estimated annual net fluxes of biological oxygen demand (BOD), total nitrogen (TN), total phosphorus (TP), faecal coliform bacteria (FC), and electric conductivity (EC) due to the water exchange between ponds and canals for a) the extensive (P1/C1), b) the semi-intensive (P2/C2), and c) the intensive aquaculture (P3/C3). Net fluxes were estimated by multiplying the average concentration difference between pond and canal water with the annual water exchange volumes reported by the farmers. One sample *t*-test results for the hypothesis that the mean flux is zero are indicated by asterisks: *p < 0.05, **p < 0.01.

Overall, aquaculture pond water quality was thus comparable (i.e. phosphorus) or better (i.e. total nitrogen, faecal coliform bacteria) than the water quality in the associated canals. An estimation of the difference between inputs and outputs in the water exchange between ponds and canals (Fig. 3) suggested that the extensive aquaculture attenuated over a year about 58.4 ± 18 kg of nitrogen pollution (or 26 ± 18 kg of nitrogen per hectare). This amounted to about 20 population equivalents of nitrogen pollution attenuation from domestic wastewater (von Sperling and Chernicharo, 2006). The semi-intensive and intensive aquacultures also appeared to attenuate nitrogen inputs from the canals, even though these observations were not statistically significant. The findings countered initial expectations that aquacultures would be significant contributors to nitrogen pollution in the canals (Ferreira et al., 2015).There is an apparent trend of reduced nitrogen pollution attenuation with intensification of the aquaculture practice, as would be expected from the reported fertilizer and feed inputs of the semi-intensive and intensive aquacultures, respectively (Table 1). Attenuation of organic nitrogen inputs from canals in the aquaculture ponds may have occurred via ammonification, ammonia volatilisation, ammonia nitrification in the pond water and aerobic surface sediment layer, and denitrification in the deeper, anaerobic sediment. In addition, the extensive aquaculture also attenuated trillions of faecal coliform bacteria inputs from the canals (Fig. 3). Attenuation of faecal coliform pollution inputs could be attributed to sunlight disinfection and poor survival of faecal coliform bacteria in the pond microbial ecosystems, similar to what has been observed in waste stabilisation ponds (von Sperling and Chernicharo, 2006).

### 3.6 Molecular microbiology data, faecal pollution and antibiotic resistance gene source tracking

**Fig. 4:**
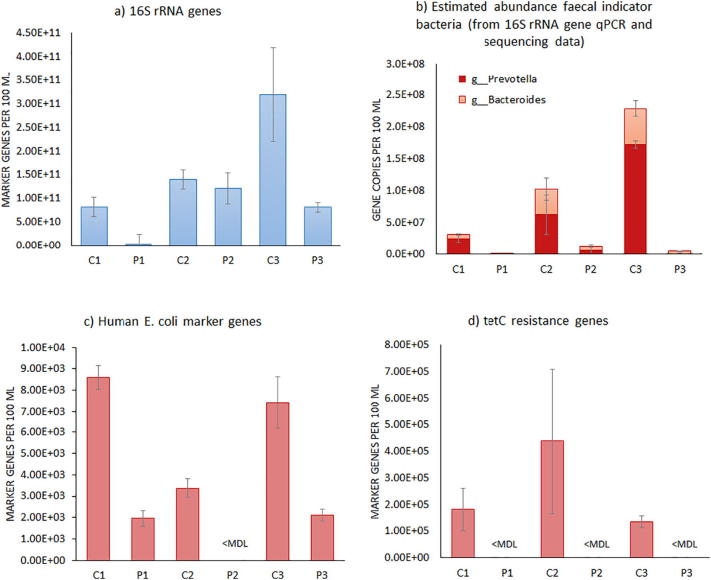
Molecular microbiology analysis of water samples from the extensive (C1/P1), semi-intensive (C2/P2) and intensive (C3/P3) aquaculture farm locations collected in June 2018. Error bars indicate the standard errors of the mean for samples from one sampling event (*n* = 2–4); <MDL = below the method detection limit.

The starkest exceedance of surface water quality criteria in the canals was observed for the faecal coliform bacteria counts which consistently exceeded 70 CFU/100 mL by orders of magnitude. For a more detailed understanding of the microbial water quality in canals and ponds, DNA was extracted from water samples during one of the sampling events (June 2018) to characterize microbial communities with molecular methods (Fig. 4).

As an indicator for the overall abundance of bacteria and archaea, 16S rRNA gene copy numbers were quantified by qPCR (Fig. 4a). They were comparable (C2/P2) or lower (C1/P1, C3/P3) in ponds as compared to the corresponding canals. Sequencing of 16S rRNA genes revealed how the canal water samples clustered at a shared Bray-Curtis similarity of 62.28% (Fig. S1a in Supporting information), while the aquaculture pond water samples diverged from this cluster. The microbial community in the extensive aquaculture (P1) was most similar (57.27%), and the microbial community of the intensive aquaculture (P3) least similar (36.67%), to canal water microbial communities. This shows that aquaculture practice shaped pond water microbiology.

At phyla level *Cyanobacteria*, *Actinobacteria*, *Proteobacteria* and *Bacteroidetes* were most frequently detected in ponds and canals (Fig. S1b in Supporting information). *Cyanobacteria* contributed between 13.3 ± 0.1% (P3) and 42.6 ± 3.6% (C3) of relative abundance. These photosynthetic bacteria can cause highly visible “algae blooms”, and had significantly higher mean relative abundance in the canals as compared to the aquaculture ponds (*t*-test, *p* < 0.01). This molecular microbiology observation aligned with the chemical indicators of greater water eutrophication in the canals.

At genera level, the mean relative abundance of 16S rRNA gene sequences which were aligned with *Bacteroides* and *Prevotella*, was overall significantly higher in canals as compared to ponds (*t*-test, *p* < 0.05 and < 0.001, respectively). *Bacteroides* and *Prevotella* are important bacterial genera in the gut microbiome of humans and other mammals (Vangay et al., 2018). Since mean total 16S rRNA gene copy numbers were overall lower in ponds as compared to the corresponding canals (*t*-test, *p* < 0.05), these data (Fig. 4b) supported the plate count findings (Fig. 2d) which already identified the canals as sources of faecal pollution into the ponds. Mean marker gene numbers for *E. coli* of human origin (Fig. 4c) were overall also significantly lower in ponds as compared to canals (*t*-test, *p* < 0.001), identifying domestic wastewater as a likely source of the faecal pollution in the canals. Only one tetracycline resistance gene (tetC) could be detected in the water samples, and was only detected in the canals (Fig. 4d). Hence, occurrence of environmental tetracycline resistance in the study area was likely caused by domestic rather than aquacultural use of tetracycline.

### 3.7 Herbicides/algaecides, pesticides and antibiotics

**Fig. 5:**
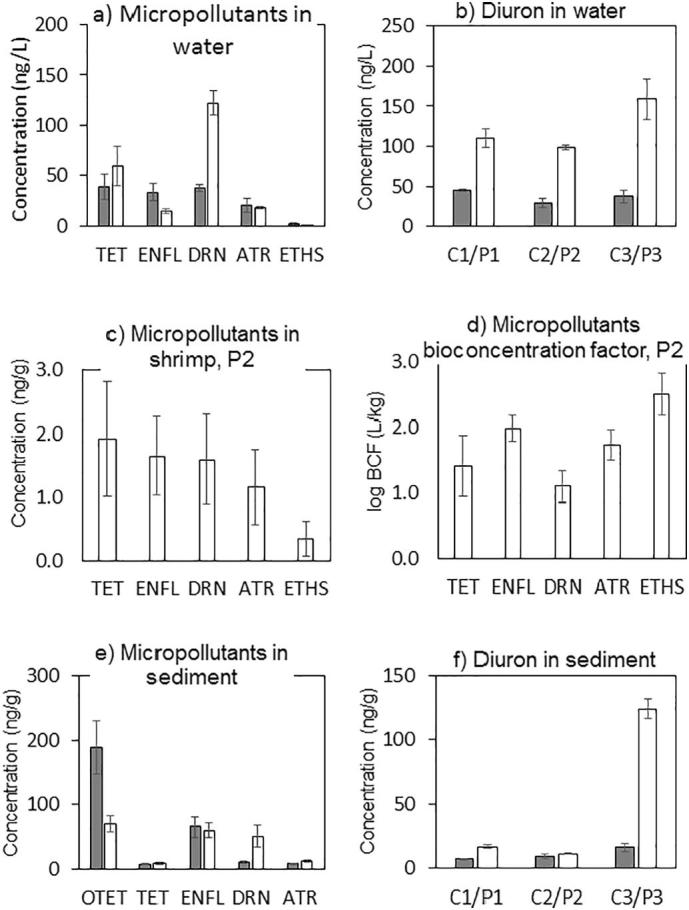
Herbicides, pesticides and antibiotics in canals (C, grey bars) and ponds (P, white bars). The mean value of all pond or canal samples are shown in plots a&e. Error bars indicate the standard error of the mean (*n* = 12, 3 locations, 4 sampling events). Plot b & f detail for diuron the mean values observed at each sampling location (*n* = 4 sampling events): for the extensive (C1/P1), semi-intensive (C2/P2) and intensive (C3/P3) aquaculture farm. Shrimp data (plot c&d) is only available for the semi-intensive farm, dry shrimp weight (P2, *n* = 9, 3 sampling events). OTET: oxytetracycline, TET: tetracycline, ENFL: enrofloxacin, DRN: diuron, ATR: atrazine, ETHS: ethoprophos. OTET concentrations in water and shrimp, and ETHS concentrations in sediment were below the detection limit.

Aquaculture farmers may have used chemicals to manage water pollution related aquaculture problems, such as algae blooms, and infectious shrimp and fish diseases. Indeed, the farmers reported use of disinfectants like lime and chlorine, insecticides, and herbicides/algaecides, but not antibiotics (Table 1). Several herbicides, pesticides and antibiotics were detected in canal and pond water samples (Fig. 5a). But concentrations measured in canals were at the lower end of those previously reported for Thai surface waters (ERTC, 2016; Rico et al., 2014; Sangchan et al., 2014). Only for diuron (Fig. 5b) were the measured concentrations statistically significantly higher in pond as compared to canal water (*t*-test, *p* < 0.001), identifying the aquacultures as sources for this herbicide/algaecide into the canals. These findings agreed with the reported diuron usage by two aquacultures (Table 1). Low concentrations of antibiotics measured in the ponds, like the antibiotic resistance data (Fig. 4d), supported statements by the farmers that they were not using antibiotics in their aquacultures.

The Thai Coastal Water Quality Standard stipulates that a range of pesticides/herbicides, including diuron and atrazine, should not be detectable in water used for aquaculture. However, given the low detection limits of modern analytical methods, a risk-based assessment would seem more appropriate, where a comparison is made between the likely exposure of organisms to a chemical and the chemical's toxicity. The calculated toxicity exposure ratios (TER) for the five micropollutants found in the water columns in this study were above the commonly used trigger value of 10. Hence, the risk of these substances to non-target organisms in surface water was currently acceptable (Table S10 in Supporting information). The combined toxicity (risk quotient) of the five micropollutants in the water column was 0.30, so the combined risk was considered also acceptable, as the risk quotient was <1.

To address concerns that the herbicides, pesticides and antibiotics detected in environmental samples may affect aquaculture produce, we also quantified these compounds in shrimps harvested from pond 2 (Fig. 5c). Several compounds were detectable in shrimp, but at low concentrations. Calculated bioconcentration factors (BCFs) ranged from 17 ± 8 (L/kg) for diuron to 548 ± 399 (L/kg) for ethoprophos (Fig. 5d). These BCF values, calculated for dry shrimp tissue concentrations, indicate low bioaccumulation potentials for the micropollutants investigated. Maximum Residue Levels (MRLs) were available for the five observed micropollutants from the European Commission and the World Health Organisation (WHO). The MRLs for animal products ranged from 0.01 to 0.05 mg/kg (Table S11 in Supporting information). Comparing these thresholds with the residues observed showed that, although these substances were present in shrimp for consumption, they were observed at a level that is acceptable to human health.

### 3.8 Canal versus pond sediment quality

Measured metal concentrations in pond sediments were below or in the same range as those measured in the associated canals (Table 3). Pond 1 sediment exceeded the Coastal Sediment Quality Guideline for arsenic, copper and zinc, pond 2 sediment exceeded the standard for arsenic, and pond 3 sediment the standards for arsenic and copper (Tables 3 and S2 in Supporting information). Total PAHs were lower in sediment from ponds as compared to sediment from the associated canals, and below Coastal Sediment Quality Guideline values of 4 mg/kg. The herbicides and antibiotics monitored in this study were not included in the Coastal Sediment Quality Guideline, but measured concentrations in sediments were nonetheless considered (Fig. 5e). Overall, the mean diuron concentration (Fig. 5e & f) was significantly higher in sediment from ponds as compared to the canals (*t*-test, *p* < 0.05), mirroring the observations for the water samples (Fig. 5a&b). Our measurements were within the range or higher (diuron in P3) as compared to previous reports for Thai sediments (Harino et al., 2006; Rico et al., 2014).

**Table 3:** Sediment quality (*n* = 3 sampling events).

Parameter	C1	C2	C3	P1	P2	P3
Arsenic (mg/kg)	14.67 ± 2.00	15.33 ± 2.52	13.37 ± 5.63	10.17 ± 5.09	9.27 ± 0.64	14.23 ± 3.96
Boron, Water Soluble (mg/kg)	3.00 ± 0.90	2.03 ± 0.23	1.93 ± 0.21	1.87 ± 0.21	1.87 ± 0.21	1.80 ± 0.36
Cadmium (mg/kg)	0.27 ± 0.15	0.10 ± 0.10	0.10 ± 0.10	0.10 ± 0.10	<0.10	0.10 ± 0.10
Chromium (mg/kg)	68.00 ± 30.35	38.00 ± 10.82	29.67 ± 6.81	25.67 ± 5.13	27.67 ± 2.12	27.67 ± 4.16
Copper (mg/kg)	127.67 ± 74.65	64.67 ± 31.79	50.33 ± 27.54	26.33 ± 2.52	20.00 ± 0.71	36.67 ± 2.08
Lead (mg/kg)	39.00 ± 3.06	43.00 ± 7.21	35.67 ± 16.04	33.00 ± 15.39	39.00 ± 3.54	33.33 ± 5.51
Mercury (mg/kg)	0.13 ± 0.07	0.09 ± 0.05	0.06 ± 0.05	<0.05	<0.05	<0.05
Nickel (mg/kg)	65.67 ± 36.67	34.00 ± 14.53	23.00 ± 10.82	16.67 ± 5.69	17.33 ± 1.41	18.00 ± 2.00
Selenium (mg/kg)	0.70 ± 0.35	0.53 ± 0.50	<0.5	<0.5	<0.5	<0.5
Zinc (mg/kg)	340.0 ± 214.9	135.6 ± 66.9	116.0 ± 42.7	110.7 ± 25.3	62.3 ± 5.0	82.7 ± 9.6
16 US EPA PAHs (mg/kg)	0.67 ± 0.11	0.22 ± 0.05	0.12 ± 018	0.06 ± 0.02	0.12 ± 0.00	0.03 ± 0.01

### 3.9 Water policy analysis

As outlined in the Introduction, Thailand has detailed regulations and guidelines for water quality requirements in coastal aquaculture. Some Thai standards are stringent in comparison with other guidance values. As discussed, the pesticide and herbicide concentrations measured in this study were below risk assessment thresholds indicating low risk to non-target organisms, and below maximum residue levels in shrimp defined by the European Commission and the WHO, but did not meet the hazard-based “not-detectable” requirements of the Thai standards for water to be used in coastal aquaculture. Also, WHO microbial water quality targets for wastewater-fed aquacultures are ≤10^4^ *E. coli*/100 mL for the protection of consumers, and ≤10^3^ *E. coli*/100 mL for the protection of aquacultural workers (WHO, 2006). The Thai standard defines a much stricter limit of 70 FC/100 mL. In reality, however, canal water in Southern Bangkok was found to be heavily polluted with faecal coliform bacteria (2*10^5^ FC/100 mL on average), and nonetheless used for aquaculture. Molecular microbiology linked this faecal pollution to human sources, raising concerns about a potential faecal-oral or faecal-dermal disease transmission pathways. In Vietnam, wastewater-fed aquacultures have been identified as risk factors for gastrointestinal (Trang et al., 2007a) and skin diseases (Trang et al., 2007b).

The algae blooms and economic losses reported by the farmers confirm that the water quality in the canals draining Bangkok is currently an impediment to peri-urban aquaculture. In this context, BMA efforts to collect more revenue via a wastewater treatment fee are to be encouraged. Sound financing is essential for expanding Bangkok's wastewater treatment infrastructures which currently serves only half of the growing city's population. The average fee per household with 5 people would be an affordable 48 baht per month (Wancharoen, 2018), which is <0.3% of the average monthly household income in Thailand (26,946 baht in 2017 (NSO, 2017)). On the other hand, a charge for the abstraction of surface water would be very costly for the family-owned aquacultures investigated in this study. Calculated annual costs based on the information in Table 1&2 and a water tax of 0.5 baht per cubic meter would be 7840, 23,533 and 21,816 baht, for the extensive, semi-intensive and intensive aquaculture, respectively. These costs would be 14 to 41 times higher than the annual household costs for the proposed wastewater treatment fee. Given that according to the Office of Agricultural Economics (OAE) aquaculture farmers in Thailand typically earn slightly less than the median national household income (OAE, 2019), such expenditures would undoubtedly exacerbate the current economic difficulties of small-scale aquacultures, and likely lead to their displacement by more lucrative land-uses in the peri-urban environment. Considering also that the main water pollution in the peri-urban case study area was identified as of urban origin, the legislative promises not to charge small farmers (Wipatayotin, 2018) are to be welcomed.

### 3.10 Outlook

In 2018, there were 35,422 ha of seawater shrimp aquacultures in Thailand, down from 48,590 ha in 2008. The reduction in ponds under culture is mainly because of issues with novel shrimp diseases, and opens up opportunities for repurposing the abandoned ponds, for example to re-establishing mangrove ecosystems, which have been severely degraded by the expansion of aquacultures (Jayanthi et al., 2019). As can be seen from space (Fig. 1), coastal aquacultures occupy a large land tract between Bangkok and the Gulf of Thailand. This coastal belt could be protected and revitalized as a vast blue-green infrastructure, providing a range of ecosystem services for the city, including flood protection, food production, biodiversity, water cleansing and recreation. Sustainable aquaculture could contribute to such ecosystem services. We estimated from the data in Fig. 3 that 35,422 ha of coastal aquacultures could potentially attenuate 314,132 population equivalents of nitrogen pollution, if managed extensively. While ponds are thus clearly unable to treat all the wastewater produced by the 69 million people living in Thailand, extensively managed coastal aquacultures and other blue-green infrastructures could, in combination with the expansion of more conventional wastewater treatment in the cities, help attenuation of urban water pollution that currently affects the Gulf of Thailand (Cheevaporn and Menasveta, 2003). However, in such a scenario, aquaculture farmers would need to be fully compensated for all the ecosystem services they provide, including flood protection, water cleansing and biodiversity, to incentivise sustainable practices. As city dwellers in Bangkok become more affluent, subsidies for the protection of peri-urban green spaces may become politically more palatable, similar to the trends observed in high income countries (Tobias, 2013). However, far-sighted land-use plans and policies would need to prevent the urban sprawl and economic difficulties from displacing extensive aquacultural activities from the peri-urban environment of Bangkok.

## 4 Conclusions

▪ Water quality in peri-urban canals did not meet the requirements set out in regulations for coastal aquaculture.
▪ Canals were typically sources of nitrogen and faecal bacteria into the aquaculture ponds.
▪ Aquaculture ponds were typically sources of salinity and the herbicide/algaecide diuron into the canals.
▪ The extensive aquaculture pond attenuated an estimate 20 population equivalent nitrogen and trillions of faecal coliform bacteria input from the canals.
▪ Water and land-use policy should encourage extensive aquaculture management practices which can contribute to faecal pollution attenuation in brackish water before it is discharged into the Gulf of Thailand.

## Declaration of competing interest

The authors declare no competing interests.

## Appendix A Supplementary data

Supplementary material Image 1
