# Supplementary material for: The food-water quality nexus in periurban aquacultures downstream of Bangkok, Thailand
Source: Sci Total Environ. 2019 Dec 10;695:133923. doi: 10.1016/j.scitotenv.2019.133923 (PMC6878219; doi:10.1016/j.scitotenv.2019.133923)
Supplement: Supplementary file 1 — Supplementary material [file mmc1.docx]

**Supplementary information**

**THE FOOD-WATER QUALITY NEXUS IN PERIURBAN AQUACULTURES DOWNSTREAM OF BANKGOK, THAILAND**

Wojciech Mrozik^†^, Soydoa Vinitnantharat^§^, Thunchanok Thongsamer^§^, Nipapun Pansuk^§^, Pavinee Pattanachan^β^, Parinda Thayanukul^£^, Kishor Acharya^†^, Marcos Quintela Baluja, Charles Hazlerigg^γ^, Aidan F. Robson^†^, Russell J. Davenport^†^, and David Werner^†,^*

^†^ School of Engineering

Newcastle University, Newcastle upon Tyne, United Kingdom

^β^ Pilot Plant Development and Training Institute

King Mongkut’s University of Technology Thonburi, Bangkok, 10140, Thailand

^§^ School of Energy, Environment and Materials

King Mongkut’s University of Technology Thonburi, Bangkok, 10140, Thailand

^£^ Department of Environmental Engineering, Faculty of Engineering

King Mongkut’s University of Technology Thonburi, Bangkok, 10140, Thailand

^γ^ Enviresearch Ltd, Newcastle upon Tyne, United Kingdom

1. **Materials and methods**

**Sampling methods.** Samples were collected on the same day from the three aquaculture ponds and their associated canals, either from on shore or using a boat/float. Several grab water samples were collected into sterilized bottles and blended to form a composite sample for each location. Sediment samples were collected with a bottom-dredge sampler. Shrimp were collected by the farmer from the semi-intensive aquaculture pond only (P2). The sampling schedule and replication is summarized in Table S4. The further processing of the samples is described for each analysis separately in the method descriptions below. **Micronutrients and metals.** Samples were filtered through a 0.22 µm PVDF syringe filter (Gilson Scientific Ltd, Dunstable UK) and collected in 15 mL polyethylene vials, with those for subsequent metals analysis acidified with lab-grade concentrated nitric acid. Analysis of filtered metals was subsequently undertaken at NU using a Varian Vista-MPX ICP-OES or Agilent 770 Series ICP-MS, as appropriate for the metal concentration. Anion analysis was conducted using a Dionex DX320 ion chromatograph. Blanks and standards were used throughout and triplicate samples analysed periodically. Metals in sediment were analysed commercially by Derwentside Environmental Testing Services Ltd., Consett, UK using accredited MCERTS methods for metals in soils.

**Molecular microbiology analysis.** Duplicate 100 mL of canal and pond water samples were filtered through 0.22 µm membranes (Sartorius UK Limited, Surrey, UK) and frozen immediately. The total DNA from bacterial biomass retained by the membrane was extracted at NU using a PowerWater® DNA Isolation Kit as per the manufacturer’s instructions (QIAGEN, Crawley, UK). DNA purity and concentration was determined using a DS-11 FX+ Spectrophotometer / Fluorometer (DeNovix, Delaware, USA).

The DNA were sequenced on the MiSeq Illumina Sequencing Platform using the protocol mentioned elsewhere ^1^ by using the primers designed by Caporaso et al. ^2^, which target the V4 region of 16S rRNA gene. For data processing, the Quantitative Insight Into Microbial Ecology (QIIME) 1.9.1 bioinfomatics pipeline (<http://www.qiime.org>) was used ^2^. Briefly, the sequences were trimmed to remove primers, linkers and adaptor, and quality filter (considering minimum quality score of 30) and chimera checks were performed with the usearch8 algorithm (Edgar, 2010). Using the UCLUST algorithm ^3^, sequences were clustered into Operational Taxonomic Unit (OTUs) at 97% identity threshold. Taxonomy assignment to OTUs was performed using the RDP naïve Bayesian rRNA classiﬁer ^4^ with the Greengenes database (http://greengenes.lbl.gov/) ^5^. In total 11,59,266 16S rRNA gene sequences were obtained, with between 53,336 and 159,723 sequence reads per sample. The QIIME generated OTU table was square root transformed and used for the diversity analysis, which includes Bray Curtis similarity metric calculation, and hierarchical cluster analysis based on group averages. The output of the Bray Curtis analysis was also used in an Analysis of Similarities (ANOSIM) which was carried out using PRIMER v7 software.

Real time PCR assays were performed to quantify the number of target genes on a BioRad CFX C1000 system (BioRad, Hercules, CA USA) using the primers shown in Table S5. For quantification of target genes, 2 μL template DNA was used in a reaction mixture containing 5 μL 2 × SsoAdvanced Universal SYBR Green Supermix (Bio-Rad), 500 nmol L^−1^ of each forward and reverse primer, and molecular grade H_2_O (Invitrogen, Life Technologies, Paisley, UK) to a final volume of 10 μL. Reaction conditions for quantification of each target gene were 98°C for 3 min (1×), then 98°C, 15 s, Primer Annealing Temperature (T_a_), 60 s (40x). All samples were run in duplicate and molecular grade H_2_O replaced template in control reactions. In order to avoid inhibitor effects, DNA samples were diluted to a working solution of 5 ng/uL.

**Micropollutant analysis.** Analysis of antibiotics, pesticides and herbicides in water and sediments was performed according to EPA method 1694. Triplicate samples were analysed for each sampling point/event. 1 L of water was passed through a 0.45 mm glass fiber filter (GF/F, Whatman, UK) and acidified with diluted HCl to pH 2.5. Next, 50 ng surrogate standards were added (enrofloxacin- D5 and atrazine-D4 from QMX Laboratories, Dunmow, UK) and left for 30 min to equilibrate. Prior to solid-phase extraction (SPE), 500 mg Na_4_EDTA·2 H_2_O was added to the sample and left for 30 min. The extraction was carried out on a Waters Oasis HLB cartridge (200mg, 6cc, USA) at KMUTT. The cartridge was conditioned with 6 mL methanol, 6 mL ultra-pure water, and 6 mL of acidified ultra-pure water (pH=2.5, HCl from Sigma-Aldrich St. Louis USA). Next, the water samples were loaded at a flow rate of 10 mL min^-1^. After extraction, the cartridges were washed with 10 mL of 5% methanol and dried for 30 min under vacuum, then transported frozen to NU. Samples were eluted with 5 mL of methanol (LCMS grade, VWR, Lutterworth, UK) and 5 mL of methanol containing 5% ammonium hydroxide (Sigma-Aldrich St. Louis USA). Next, samples were concentrated to complete dryness at 35ºC under a gentle stream of nitrogen in the Vertex evaporator (Labconco, USA). Finally, they were reconstituted in a 1 mL of the mobile phase, ready for LC-MS analysis.

Sediment samples were air dried, and then ground, freeze-dried and sieved (<212 mesh) at NU. Next, 2g of each sample (triplicates) were spiked with 50 ng of surrogate standards and put into a 50 mL Falcon tube. Twenty millilitres of acetonitrile (LCMS grade, VWR, Lutterworth, UK) and 15 mL of phosphate buffer (Sigma-Aldrich St. Louis USA) were added to the solid sample, sonicated for 30 min, and centrifuged for 10 min at 4500 rpm. The supernatant was then decanted into a round-bottom flask. This procedure was repeated three times. The combined supernatant was evaporated on a rotary evaporator and finally diluted with deionized water to 500 mL. Next, the same SPE procedure as for the water samples was applied.

The freeze-dried shrimps were extracted with a previously tested protocol ^6^. At KMUTT, samples were homogenized and 2 g of each shrimp (triplicates) were placed into a Falcon tube. Then samples were spiked with 50 ng of surrogate standards and equilibrated for 30 min. Next, EDTA solution (1 mL of 0.1 M) and 40 ml of extraction buffer (acetonitrile:citric buffer, 80:20) were added. Samples were vortexed for 10 min and put into a sonic bath (ultrasonic cleaner, model 690HT, CREST, USA) for 20 min. After sonification, samples were centrifuged for 10 min at 4500 rpm. The supernatant was then decanted into a round-bottom flask. The procedure was repeated three times. The combined supernatant was evaporated on the rotary evaporator and finally diluted with deionized water to 500 mL. Next, the same SPE procedure as for the water samples was applied.

The selected micropollutants were analysed by a UPLC-MS/MS system at NU. As the chromatograph the Acquity UPLC Classic was used (Waters, Elstree, UK) equipped with a ACE C-18 PFP column (2.1x100 mm, 1.7 um, HiChrom, Theale, UK). The column was thermostated at 40 ºC during the runs. The mobile phase consisted of A (water with 0.1% formic acid) and B (acetonitrile with 0.1% formic acid). The flow rate was of 0.4 mL/min. The gradient program was applied as follows: 0-0.25 min: 10% B; 0.25-9.5 min: 98% B; 9.5-10.5 min: 98% B; 10.5-11.5 min: 10% B; 11.5-14 min: 10% B (equilibration between samples).

A Triple Quad Waters Xevo – TQS mass analyser (Waters, Elstree, UK) was used for MS/MS analysis. The system was equipped with an ESI source and operated in positive ionization mode. The operating parameters were: capillary voltage, 3.0 kV; source temperature, 150 °C; desolvation temperature, 600 °C; cone gas flow, 150 L h^−1^; desolvation gas, 1000 L h^−1^; collision gas, 0.15 mL min^-1^ and nebuliser gas, 7.00 bar. Quantification of each target compound was performed in MRM mode. MS/MS parameters are listed in Table S6 in supporting information. All data were acquired and processed using MassLynx 4.1 software (Waters, Elstree, UK). Validation and quality control parameters are listed in Table S3 in supporting information.

For the analysis of 16 US EPA PAHs, 5 g of sediment (duplicates) were extracted by accelerated solvent extraction (ASE, ThermoFisher Scientific, Waltham, USA) using hexane:acetone (pesticide grade from Sigma-Aldrich, St. Louis, US) 50:50 v:v. Sample clean-up and analysis followed protocols described by Hale et al. ^7^.

**Risk assessment for micropollutants.** A risk-based assessment for surface water (e.g. freshwater edge-of-field streams, ditches and ponds) generally takes the form of a Toxicity Exposure Ratio:

TER=Toxicity/Exposure

where the toxicity is the concentration that causes 50% effect (EC50) in acute studies or No Observed Effect (NOEC) in chronic studies as determined from laboratory tests, and the exposure is the concentration in the environment from the use of the chemical product. This TER approach was used with known substance-specific toxicity data available in the literature, environmental concentrations observed in the tested waterbodies and a trigger value of 10. Combination toxicity was also assessed using a simple additive model based on the following equation:

$$CT=\sum\left( \frac{Trigger}{TER} \right)_{i}+\left( \frac{Trigger}{TER} \right)_{j}+\ldots\left( \frac{Trigger}{TER} \right)_{n}$$

where i, j and n are different chemicals present in the water column. For the risk to non-target organisms to be acceptable, the combined risk value must be below 1.

**Questionnaire.** The following questionnaire was used to interview the three aquaculture farm owners (translated from Thai).

**Name of KMUTT researcher………………………………………Date……………….……**

**Farm Location…………………………………………….………………………….……**

O Extensive farm O Semi-intensive farm O Intensive farm

1. **General Information**

**Farm owner**

Age O 25-35 O 35-45 O 45-55 O > 55

O Male O Female Working Years………………………

Type of aquatic animal ………………………….…Pond area……………………Pond depth………

Size ………………Numbers of stock added………….……………………

O adding new stock during culture O not adding new stock during culture

If adding, How often **………………………….**numbers of stock added **……………………**

**2 Activities at farm**

**2.1 Pond preparation**

Date of pond preparation……………………………………………………

**O Aeration**

Time duration .............................................

Note...........................................................

**O Chemical adding**

Type of chemical/biological uses (Lime, antibiotics, fertilizer, etc.)

1. Lime O Yes O No Name...................Dosage use............................Frequency.........................

2. Fertilizer O Yes O No Name...................Dosage use............................Frequency.........................

3. Color O Yes O No Name..................Dosage use............................Frequency........................

4. Antibiotics O Yes O No Name.................Dosage use............................Frequency...........................

5. Probiotics O Yes O No Name.................Dosage use............................Frequency...........................

6. Herbicides O Yes O No Name.................Dosage use............................Frequency...........................

7. Others O Yes O No Name.................Dosage use............................Frequency...........................

8. Others O Yes O No Name.................Dosage use............................Frequency...........................

Do you exchange the water? O Yes O No

If yes, when .........................................

The volume of water or percentage of water drain out .........................

Note:.............................................................................................................................................................

..................................................................................................................................................................

**2.2 Culture**

O Water Quality problem in each season (problem and solution, specify the type and amount of chemicals if used)

Wet season

1. Disease outbreak.............................solved by...........................................................

.....................................................................................................................................

2. Fish Dead .................................... solved by..............................................................

..........................................................................................................................................

3. Algae bloom...................................... solved by............................................................

...........................................................................................................................................

4. High rainfall...................................... solved by............................................................

................................................................ ..........................................................................

5............................................................. solved by.............................................................

...........................................................................................................................................

Dry season

1. Disease outbreak.............................solved by...........................................................

.....................................................................................................................................

2. Fish Dead .................................... solved by..............................................................

..........................................................................................................................................

3. Algae bloom...................................... solved by............................................................

...........................................................................................................................................

4. . ................... ..................................... solved by............................................................

................................................................ ..........................................................................

5............................................................. solved by.............................................................

...........................................................................................................................................

O Water Exchange

Time to drain out water from pond.......................Volume of water or % water drained........................

Frequency..................................................

Note..................................................................................................................

O Aeration

O day : location & motor ............................................... time duration.......................................

O night: location & motor ............................................... time duration........................................

Note......................................................................................................................................................

O Feed : Feed name.......................... amount of feed used (kg) ..........................

frequency...................................Note. ...........................................................................................

O Lime/Calcium carbonate name.......................... amount of lime used (kg) ..........................

frequency...................................Note. ...........................................................................................

O Zeolite name.......................... amount of zeolite used (kg) ..........................

frequency...................................Note. ...........................................................................................

O Disease control: Chemical Name.............................amount used (kg)................

frequency...................................Note. ...........................................................................................

O Minerals: Chemical Name.............................amount used (kg)................

frequency...................................Note. ...........................................................................................

O Fertilizer: Chemical Name.............................amount used (kg)................

frequency...................................Note. ...........................................................................................

O Herbicides: Chemical Name.............................amount used (kg)................

frequency...................................Note. ...........................................................................................

O Probiotics: Chemical Name.............................amount used (kg)................

frequency...................................Note. ...........................................................................................

O Others: Chemical Name.............................amount used (kg)................

frequency...................................Note. ...........................................................................................

Note............................................................................................................................................................ O Other activities in each season

...............................................................................................................................................................................................................................................................................................................................................................................................................................................................................................................................................................................................................

**2.3 Harvest and Management**

Harvest

Numbers of fish or shrimp .......................Weight ........................kg

How to harvest............................................................................

Volume of water drained out.....................Type of water pump ....................time ....................

Management

Shrimp market.............................................

Sediment management (dredging/use of sediment/)…………………………………………

**Table S1**: Coastal Water Quality Standard. Class 3: Aquaculture areas shall be applied to the water that is designated by fisheries laws to be used for aquacultural practices. Source: Pollution Control Department by Notification of the National Environment Board (2007).

| **Parameter** | **Units** | **Standard Value for Class 3** |
| --- | --- | --- |
| Floatable Solids | – | not objectionable |
| Floatable Oil & Grease | – | invisible |
| Colour | Forel-Ule | 1.0-22.0 |
| Odour | – | not objectionable |
| Temperature | (C°) | an increase shall not more than 1 C° from the natural temperature |
| pH | – | 7.0-8.5 |
| Transparency | m | a decrease shall not be more than 10% of the minimum transparency governed by natural condition |
| Suspended Solids | mg/l | an increase shall not more than the average value within 1 day, 1 month or 1 year added by its corresponding deviation value |
| Salinity | ppt | any change shall not be more than 10% of the minimum salinity |
| Petroleum Hydrocarbon | ug/l | ≤ 0.5 |
| Dissolved Oxygen | mg/l | ≥ 4 |
| Total Coliform Bacteria (MPN/100 ml) | MPN/100 ml | ≤ 1,000 |
| Fecal Coliform Bacteria (CFU/100 ml) | CFU/100 ml | ≤ 70 |
| Enterococci Bacteria (CFU/100 ml) | CFU/100 ml | - |
| Nitrate -Nitrogen | ug-N/l | ≤ 60 |
| Phosphate-Phosphorus | ug-P/l | ≤ 45 |
| Unionized Ammonia | ug-N/l | ≤ 100 |
| Total Mercury | ug/l | ≤ 0.1 |
| Cadmium | ug/l | ≤ 5 |
| Total Chromium | ug/l | ≤ 100 |
| Chromium Hexavalent | ug/l | ≤ 50 |
| Lead | ug/l | ≤ 8.5 |
| Copper | ug/l | ≤ 8 |
| Manganese | ug/l | ≤ 100 |
| Zinc | ug/l | ≤ 50 |
| Iron | ug/l | ≤ 300 |
| Arsenic | ug/l | ≤ 10 |
| Fluoride | mg/l | ≤ 1 |

**Table S1 continued**:

| **Parameter** | **Units** | **Standard Value for Class 3** |
| --- | --- | --- |
| Residual Chlorine | mg/l | - |
| Phenol | mg/l | ≤ 0.03 |
| Sulfide | ug/l | ≤ 10 |
| Cyanide | ug/l | ≤ 7 |
| Polychlorinated Biphenyl, PCBs | ug/l | Non-detectable |
| Tributyltin | ng/l | ≤ 10 |
| Radioactivity - alpha - beta (excluding potassium 40) | Bq/l | ≤ 0.1 ≤ 1 |
| Containing-chlorine pesticides - Aldrin - Chlordane - DDT - Dieldrin - Endrin - Endosulfan - Heptachlor - Lindane | ug/l | ≤ 1.3 ≤ 0.004 ≤ 0.001 ≤ 0.0019 ≤ 0.0023 ≤ 0.0087 ≤ 0.0036 ≤ 0.16 |
| Other pesticides - Alachlor - Ametryn - Atrazine - Carbaryl - Carbendazim - Chlorpyrifos - Cypermethrin - 2,4-D - Diuron - Glyphosate - Malathion - Mancozeb - Methyl Parathion - Parathion - Propanil | ug/l | Non-detectable |

**Table S2**: Coastal Sediment Quality Guideline (announced by the Pollution Control Department on 9 October 2015).

| **Parameters** | **Unit (per kg DW)** | **Maximum Permitted Values** |
| --- | --- | --- |
| Cadmium | mg/kg DW | 2 |
| Chromium | mg/kg DW | 42 |
| Lead | mg/kg DW | 52 |
| Copper | mg/kg DW | 25 |
| Mercury | mg/kg DW | 0.4 |
| Zinc | mg/kg DW | 102 |
| Arsenic | mg/kg DW | 7 |
| Chlordane | ug/kg DW | 3 |
| Dieldrin | ug/kg DW | 0.8 |
| DDT | ug/kg DW | 0.6 |
| Heptachlor | ug/kg DW | 11 |
| Total PAHs: TPAHs | ug/kg DW | 4,000 |
| Low Molecular Weight PAHs: LPAHs | ug/kg DW | 550 |
| High Molecular Weight PAHs: HPAHs | ug/kg DW | 1,700 |
| Polychlorinated biphenyls: PCBs | ug/kg DW | 23 |
| Tributyltin: TBT | ug/kg DW | 5,500 |

**Table S3**: Effluent Quality Standard for Coastal Aquaculture for ponds larger than 10 rai (16,000 m^2^). Notification of the Ministry of Natural Resources and Environment (2004).

| **Parameter** | **Unit** | **Range or Maximum Permitted Values** |
| --- | --- | --- |
| pH | - | 6.5-9.0 |
| Biochemical Oxygen Demand | mg/l | 20.0 |
| Suspended Solids | mg/l | 70.0 |
| NH_3_ –N | mg-N/l | 1.1 |
| H_2_S | mg/l | 0.01 |
| Total Phosphorus | mg-P/l | 0.4 |
| Total Nitrogen | mg-N/l | 4.0 |

**Table S4:** Sampling schedule, replication and parameters analysed

| Sample type | Sampling locations | Sampling events | Replicates per sampling event | Parameters analysed |
| --- | --- | --- | --- | --- |
| Water | P1, C1, P2, C2, P3, C3 | March 17, June 17, July 17, Aug 17, Sept 17, Oct 17, Dec 17, Jan 18 | 1 | pH, dissolved oxygen, electrical conductivity, temperature, biochemical oxygen demand, chemical oxygen demand, nitrite, nitrate, ammonia, ortho-phosphate phosphorus, total phosphorus, suspended solids, fat, oil and grease |
| Water | P1, C1, P2, C2, P3, C3 | June 17, July 17, Aug 17, Dec 17, Jan 18, Feb 18 | 1 | Fecal coliform bacteria |
| Water | P1, C1, P2, C2, P3, C3 | April 2017, June 2018 | 1 | Metals (Ca, Mg, Na, K, Ba, Fe, Mn, Al, Zn, Pb, Cu, As, Cd, Sb, Si, Ni, Cr, Sr) |
| Water | P1, C1, P2, C2, P3, C3 | March 2017, Jan 2018, May 2018, June 2018 | 3 | Oxytetracycline, tetracycline, enrofloxacin, ethoprophos, atrazine, diuron |
| Water | P1, C1, P2, C2, P3, C3 | June 2018 | 2 | 16S rRNA amplicon sequencing, marker genes for total bacteria, Hu 8, Hu 100, tetB, tetC, tetO, tetQ, tetT, tetW, tetX |
| Sediment | P1, C1, P2, C2, P3, C3 | March 2017, Jan 2018, May 2018, June 2018 | 3 | Oxytetracycline, tetracycline, enrofloxacin, ethoprophos, atrazine, diuron |
| Sediment | P1, C1, P2, C2, P3, C3 | March 2017, Jan 2018, May 2018 | 1 | Metals (As, B, Ca, Cr, Cu, Pb, Hg, Ni, Se, Zn), PAHs |
| Shrimp | P2 | Jan 2018, May 2018, June 2018 | 3 | Oxytetracycline, tetracycline, enrofloxacin, ethoprophos, atrazine, diuron |

**Table S5:** Real-time qPCR primers

| Target Organisms | Primer | Sequence (5'>>>3') | Amp-licon size | Reference |
| --- | --- | --- | --- | --- |
| Human *E.coli* | Hu 8 -F | ACAGTCAGCGAGATTCTTC | 177 | ^8^ |
|  | Hu 8 -R | GAACGTCAGCACCACCAA |  |  |
|  | Hu 100 –F | ACGGTTATCAGCTCACGTCG | 98 | (Robson and Davenport, in preparation) |
|  | Hu 100 -R | TCGCCCCTCGAAAAGCATTA |  |  |
| Total Bacteria (16S rRNA) | 1055 F | ATGGCTGTCGTCAGCT | 337 | ^9^ |
|  | 1392 R | ACGGGCGGTGTGTAC |  |  |
| Tetracycline resistance | tetB F | AAAACTTATTATATTATAGTC | 167 | ^10^ |
|  | tetB R | TGGAGTATCAATAATATTCAC |  |  |
|  | tetC F | GCGGGATATCGTCCATTCCG | 207 |  |
|  | tetC R | GCGTAGAGGATCCACAGGACG |  |  |
|  | tetO F | ATGTGGATACTACAACGCATGAGATT | 101 |  |
|  | tetO R | TGCCTCCACATGATATTTTTCCT |  |  |
|  | tetQ F | AGAATCTGCTGTTTGCCAGTG | 169 |  |
|  | tetQ R | CGGAGTGTCAATGATATTGCA |  |  |
|  | tetT F | AAGGTTTATTATATAAAAGTG | 167 |  |
|  | tetT R | AGGTGTATCTATGATATTTAC |  |  |
|  | tetW F | GAGAGCCTGCTATATGCCAGC | 168 |  |
|  | tetW R | GGGCGTATCCACAATGTTAAC |  |  |
|  | tetX F | CAATAATTGGTGGTGGACCC | 468 |  |
|  | tetX R | TTCTTACCTTGGACATCCCG |  |  |

**Table S6:** Optimized MS/MS parameters for the analysis of veterinary drugs, pesticides and herbicides

| Analytes | Abbreviation | Parent ion  (m/z) | Daughter ion  (m/z) | Fragmentor  (V) | Collision Energy  (eV) |
| --- | --- | --- | --- | --- | --- |
| Oxytetracycline | OTC | 460 | 426  381 | 20  20 | 16  20 |
| Tetracycline | TC | 444 | 410  427 | 25  25 | 19  13 |
| Enrofloxacin | ENFL | 359 | 342  316 | 20  20 | 18  25 |
| Enrofloxacin d5 | ENFL d5 | 364 | 321 | 20 | 18 |
| Ethoprophos | ETHS | 242 | 131  97.2 | 22  22 | 29  18 |
| Atrazine | ATR | 215 | 174.2  96.2 | 22  22 | 16  34 |
| Atrazine d5 | ATR d5 | 220 | 179 | 22 | 15 |
| Diuron | DRN | 232 | 72.2  46.2 | 22  22 | 16  13 |

**Table S7:** Validation and quality control parameters for the analysis of veterinary drugs, pesticides and herbicides. W = Water, Sed = Sediment, Shr = Shrimp.

| Analyte | Surrogate | Recovery (%) | | | LOD (n=5) | | | LOQ (n=5) | | | |
| --- | --- | --- | --- | --- | --- | --- | --- | --- | --- | --- | --- |
|  |  | W  ng/ L | Sed  ng/g | Shr ng/g | W  ng/ L | Sed  ng/g | Shr ng/g | | W ng/L | Sed  ng/g | Shr  ng/g |
| OTC | ENFL d5 | 90±7.2 | 62±10.3 | 109.2±8.2 | 0.3 | 0.5 | 0.6 | | 1.1 | 1.6 | 2.0 |
| TC | ENFL d5 | 102±6.5 | 72±7. | 103.0±1.6 | 0.3 | 0.4 | 0.5 | | 1.1 | 1.4 | 1.5 |
| ENFL | ENFL d5 | 97±9.1 | 75.5±10.1 | 85.2±7.0 | 0.4 | 0.6 | 2.3 | | 1.2 | 1.7 | 7.0 |
| ETHS | ATR d5 | 66±6.1 | 71±8.1 | 62±9.8 | 0.1 | 0.2 | 0.21 | | 0.4 | 0.8 | 0.8 |
| ATR | ATR d5 | 95±2.6 | 105±5.3 | 78±4.1 | 0.06 | 0.07 | 0.09 | | 0.2 | 0.25 | 0.3 |
| DRN | ATR d5 | 110±4.9 | 99±3.1 | 81±5.2 | 0.06 | 0.08 | 0.08 | | 0.18 | 0.025 | 0.25 |

**Table S8**: General water quality indicators in canals (C) and ponds (P) for the extensive (C1/P1), semi-intensive (C2/P2) and intensive (C3/P3) aquaculture farm. Red: Not meeting Coastal Water Quality Standard for Aquaculture (CWQS Table S1). Orange: Not meeting Effluent Quality Standard for Coastal Aquaculture (FEQS Table S3).

| **Dissolved oxygen (mg/L) CWQS ≥ 4 mg/L** | | | | | | |
| --- | --- | --- | --- | --- | --- | --- |
| Date | C1 | C2 | C3 | P1 | P2 | P3 |
| 29.3.17 | 1.67 | 5.58 | 6.66 | 4.35 | 4.99 | 6.71 |
| 21.6.17 | 5.66 | 3.35 | 4.75 | 5.82 | 4.89 | 4.60 |
| 19.7.17 | 4.15 | 1.81 | 4.41 | 5.65 | 3.79 | 8.14 |
| 15.8.17 | 2.60 | 2.19 | 3.57 | 4.10 | 4.96 | 5.37 |
| 12.9.17 | 4.01 | 2.67 | 5.56 | 5.34 | 3.83 | 2.63 |
| 12.10.17 | 3.29 | 2.84 | 3.72 | 3.51 | 4.13 | 3.22 |
| 20.12.17 | 2.53 | 3.77 | 3.76 | 3.04 | 3.38 | 2.32 |
| 12.1.18 | 3.50 | 1.38 | 2.18 | 1.24 | 2.59 | 2.80 |
| min | 1.67 | 1.38 | 2.18 | 1.24 | 2.59 | 2.32 |
| max | 5.66 | 5.58 | 6.66 | 5.82 | 4.99 | 8.14 |
| Mean | 3.43 | 2.95 | 4.33 | 4.13 | 4.07 | 4.47 |
| STDE | 0.43 | 0.47 | 0.48 | 0.54 | 0.30 | 0.75 |
| STD | 1.22 | 1.32 | 1.36 | 1.54 | 0.85 | 2.13 |
| **Biological oxygen demand (mg/L) FEQS < 20 mg/L** | | | | | | |
| Date | C1 | C2 | C3 | P1 | P2 | P3 |
| 29.3.17 | 23.33 | 21.00 | 6.17 | 6.00 | 12.83 | 12.83 |
| 21.6.17 | 22.83 | 16.33 | 8.50 | 11.50 | 15.33 | 12.50 |
| 19.7.17 | 13.13 | 12.75 | 7.75 | 3.00 | 8.00 | 9.88 |
| 15.8.17 | 14.38 | 11.88 | 12.88 | 9.00 | 16.38 | 9.13 |
| 12.9.17 | 8.00 | 6.63 | 7.13 | 6.00 | 5.63 | 4.13 |
| 12.10.17 | 2.50 | 2.88 | 2.63 | 3.13 | 6.25 | 2.12 |
| 20.12.17 | 4.38 | 7.38 | 3.00 | 7.63 | 7.75 | 5.25 |
| 12.1.18 | 11.50 | 6.50 | 9.38 | 8.63 | 6.88 | 7.25 |
| min | 2.50 | 2.88 | 2.63 | 3.00 | 5.63 | 2.12 |
| max | 23.33 | 21.00 | 12.88 | 11.50 | 16.38 | 12.83 |
| Mean | 12.51 | 10.67 | 7.18 | 6.86 | 9.88 | 7.89 |
| STDE | 2.72 | 2.11 | 1.18 | 1.04 | 1.52 | 1.38 |
| STD | 7.70 | 5.98 | 3.35 | 2.93 | 4.29 | 3.89 |
| **Chemical oxygen demand (mg/L)** | | | | | | |
| Date | C1 | C2 | C3 | P1 | P2 | P3 |
| 29.3.17 | 57.12 | 19.99 | 34.27 | 62.83 | 102.82 | 102.82 |
| 21.6.17 | 45.70 | 45.70 | 74.26 | 45.70 | 68.54 | 57.12 |
| 19.7.17 | 40.00 | 40.00 | 48.00 | 48.00 | 40.00 | 48.00 |
| 15.8.17 | 24.00 | 64.00 | 88.00 | 96.00 | 80.00 | 64.00 |
| 12.9.17 | 96.00 | 72.00 | 56.00 | 56.00 | 64.00 | 80.00 |
| 12.10.17 | 88.00 | 80.00 | 64.00 | 56.00 | 112.00 | 64.00 |
| 20.12.17 | 112.00 | 136.00 | 144.00 | 80.00 | 112.00 | 112.00 |
| 12.1.18 | 24.00 | 48.00 | 56.00 | 48.00 | 64.00 | 64.00 |
| min | 24.00 | 19.99 | 34.27 | 45.70 | 40.00 | 48.00 |
| max | 112.00 | 136.00 | 144.00 | 96.00 | 112.00 | 112.00 |
| Mean | 60.85 | 63.21 | 70.57 | 61.57 | 80.42 | 73.99 |
| STDE | 11.94 | 12.39 | 11.96 | 6.28 | 9.27 | 7.99 |
| STD | 33.77 | 35.06 | 33.81 | 17.76 | 26.21 | 22.59 |

**Table S8 continued**: General water quality indicators in canals (C) and ponds (P) for the extensive (C1/P1), semi-intensive (C2/P2) and intensive (C3/P3) aquaculture farm. Red: Not meeting Coastal Water Quality Standard for Aquaculture (CWQS Table S1). Orange: Not meeting Effluent Quality Standard for Coastal Aquaculture (FEQS Table S3).

| **Fat/oil/grease (mg/L)** | | | | | | |
| --- | --- | --- | --- | --- | --- | --- |
| Date | C1 | C2 | C3 | P1 | P2 | P3 |
| 29.3.17 | 0.01 | 1.46 | 1.74 | 0.01 | 3.64 | 3.64 |
| 21.6.17 | 0.10 | 4.18 | 0.13 | 0.12 | 0.19 | 0.33 |
| 19.7.17 | 1.21 | 0.03 | 0.07 | 0.07 | 3.14 | 0.37 |
| 15.8.17 | 0.02 | 0.02 | 0.26 | 0.02 | 0.01 | 0.02 |
| 12.9.17 | 1.16 | 0.44 | 0.48 | 0.66 | 0.74 | 0.78 |
| 12.10.17 | 0.58 | 0.26 | 1.05 | 0.02 | 0.52 | 1.02 |
| 20.12.17 | 0.09 | 0.06 | 0.06 | 0.08 | 0.06 | 0.02 |
| 12.1.18 | 0.55 | 0.44 | 0.70 | 0.48 | 0.14 | 0.32 |
| min | 0.01 | 0.02 | 0.06 | 0.01 | 0.01 | 0.02 |
| max | 1.21 | 4.18 | 1.74 | 0.66 | 3.64 | 3.64 |
| Mean | 0.47 | 0.86 | 0.56 | 0.18 | 1.05 | 0.81 |
| STDE | 0.18 | 0.50 | 0.21 | 0.09 | 0.52 | 0.42 |
| STD | 0.50 | 1.42 | 0.59 | 0.25 | 1.47 | 1.19 |
| **Total Kjeldahl Nitrogen (mg/L)** | | | | | | |
| Date | C1 | C2 | C3 | P1 | P2 | P3 |
| 29.3.17 | 7.97 | 11.40 | 1.67 | 0.69 | 3.77 | 3.77 |
| 21.6.17 | 7.84 | 6.16 | 1.89 | 1.12 | 1.96 | 2.38 |
| 19.7.17 |  |  |  |  |  |  |
| 15.8.17 | 5.45 | 5.73 | 2.09 | 0.90 | 2.37 | 1.67 |
| 12.9.17 | 3.00 | 2.86 | 4.47 | 1.60 | 2.09 | 2.10 |
| 12.10.17 | 4.20 | 4.41 | 1.96 | 3.64 | 2.45 | 2.87 |
| 20.12.17 | 1.61 | 3.29 | 7.70 | 1.75 | 3.92 | 1.33 |
| 12.1.18 | 5.60 | 1.82 | 4.34 | 0.14 | 2.59 | 3.85 |
| min | 1.61 | 1.82 | 1.67 | 0.14 | 1.96 | 1.33 |
| max | 7.97 | 11.40 | 7.70 | 3.64 | 3.92 | 3.85 |
| Mean | 5.09 | 5.09 | 3.44 | 1.40 | 2.73 | 2.57 |
| STDE | 0.89 | 1.20 | 0.84 | 0.43 | 0.30 | 0.37 |
| STD | 2.36 | 3.18 | 2.22 | 1.13 | 0.79 | 0.98 |
| **Ammonia-Nitrogen (mg/L) FEQS < 1.1 mg/L** | | | | | | |
| Date | C1 | C2 | C3 | P1 | P2 | P3 |
| 29.3.17 | 0.275 | 0.216 | 0.060 | 0.019 | 0.040 | 0.040 |
| 21.6.17 | 0.128 | 0.121 | 0.044 | 0.025 | 0.020 | 0.036 |
| 19.7.17 | 0.175 | 0.237 | 0.131 | 0.070 | 0.009 | 0.026 |
| 15.8.17 | 0.231 | 0.152 | 0.138 | 0.006 | 0.009 | 0.011 |
| 12.9.17 | 0.080 | 0.035 | 0.014 | 0.004 | 0.008 | 0.056 |
| 12.10.17 | 0.027 | 0.031 | 0.020 | 0.021 | 0.016 | 0.015 |
| 20.12.17 | 0.049 | 0.016 | 0.123 | 0.145 | 0.122 | 0.006 |
| 12.1.18 | 0.159 | 0.020 | 0.162 | 0.042 | 0.013 | 0.082 |
| min | 0.027 | 0.016 | 0.014 | 0.004 | 0.008 | 0.006 |
| max | 0.275 | 0.237 | 0.162 | 0.145 | 0.122 | 0.082 |
| Mean | 0.141 | 0.104 | 0.087 | 0.041 | 0.029 | 0.034 |
| STDE | 0.031 | 0.032 | 0.021 | 0.017 | 0.014 | 0.009 |
| STD | 0.087 | 0.091 | 0.058 | 0.047 | 0.039 | 0.026 |

**Table S8 continued**: General water quality indicators in canals (C) and ponds (P) for the extensive (C1/P1), semi-intensive (C2/P2) and intensive (C3/P3) aquaculture farm. Red: Not meeting Coastal Water Quality Standard for Aquaculture (CWQS Table S1). Orange: Not meeting Effluent Quality Standard for Coastal Aquaculture (FEQS Table S3).

| **Nitrite-Nitrogen (mg/L)** | | | | | | |
| --- | --- | --- | --- | --- | --- | --- |
| Date | C1 | C2 | C3 | P1 | P2 | P3 |
| 29.3.17 | 0.010 | 0.020 | 0.352 | 0.005 | 0.006 | 0.006 |
| 21.6.17 | 0.071 | 0.081 | 0.074 | 0.003 | 0.006 | 1.346 |
| 19.7.17 | 0.032 | 0.018 | 0.053 | 0.005 | 0.005 | 0.005 |
| 15.8.17 | 0.137 | 0.111 | 0.091 | 0.004 | 0.004 | 0.040 |
| 12.9.17 | 0.079 | 0.138 | 0.066 | 0.020 | 0.014 | 0.541 |
| 12.10.17 | 0.108 | 0.102 | 0.160 | 0.035 | 0.023 | 0.084 |
| 20.12.17 | 0.071 | 0.023 | 0.301 | 0.088 | 0.165 | 0.109 |
| 12.1.18 | 0.012 | 0.036 | 0.012 | 0.002 | 0.056 | 0.097 |
| min | 0.010 | 0.018 | 0.012 | 0.002 | 0.004 | 0.005 |
| max | 0.137 | 0.138 | 0.352 | 0.088 | 0.165 | 1.346 |
| Mean | 0.065 | 0.066 | 0.139 | 0.020 | 0.035 | 0.278 |
| STDE | 0.016 | 0.017 | 0.044 | 0.011 | 0.020 | 0.164 |
| STD | 0.045 | 0.048 | 0.124 | 0.030 | 0.055 | 0.465 |
| **Nitrate-Nitrogen (mg/L) CWQS ≤ 0.060 mg/L** | | | | | | |
| Date | C1 | C2 | C3 | P1 | P2 | P3 |
| 29.3.17 | 0.015 | 0.007 | 0.041 | 0.017 | 0.014 | 0.014 |
| 21.6.17 | 0.031 | 0.033 | 0.031 | 0.034 | 0.048 | 0.237 |
| 19.7.17 | 0.038 | 0.072 | 0.042 | 0.025 | 0.037 | 0.034 |
| 15.8.17 | 0.012 | 0.007 | 0.013 | 0.007 | 0.019 | 0.016 |
| 12.9.17 | 0.019 | 0.012 | 0.011 | 0.015 | 0.007 | 0.061 |
| 12.10.17 | 0.067 | 0.069 | 0.066 | 0.075 | 0.069 | 0.018 |
| 20.12.17 | 0.032 | 0.011 | 0.040 | 0.084 | 0.032 | 0.660 |
| 12.1.18 | N.D. | 0.004 | N.D. | 0.031 | 0.006 | 0.691 |
| min | 0.012 | 0.004 | 0.011 | 0.007 | 0.006 | 0.014 |
| max | 0.067 | 0.072 | 0.066 | 0.084 | 0.069 | 0.691 |
| Mean | 0.031 | 0.027 | 0.035 | 0.036 | 0.029 | 0.216 |
| STDE | 0.007 | 0.010 | 0.007 | 0.010 | 0.008 | 0.104 |
| STD | 0.019 | 0.028 | 0.019 | 0.028 | 0.022 | 0.293 |
| **Total Nitrogen (calculated) (mg/L) FEQS < 4.0 mg/L** | | | | | | |
| Date | C1 | C2 | C3 | P1 | P2 | P3 |
| 29.3.17 | 7.99 | 11.42 | 2.06 | 0.71 | 3.79 | 3.79 |
| 21.6.17 | 7.94 | 6.27 | 1.99 | 1.16 | 2.01 | 3.96 |
| 19.7.17 |  |  |  |  |  |  |
| 15.8.17 | 5.60 | 5.84 | 2.19 | 0.91 | 2.39 | 1.72 |
| 12.9.17 | 3.09 | 3.01 | 4.54 | 1.63 | 2.11 | 2.70 |
| 12.10.17 | 4.37 | 4.58 | 2.19 | 3.75 | 2.54 | 2.97 |
| 20.12.17 | 1.71 | 3.32 | 8.04 | 1.92 | 4.12 | 2.10 |
| 12.1.18 | 5.61 | 1.86 | 4.35 | 0.17 | 2.65 | 4.64 |
| min | 1.71 | 1.86 | 1.99 | 0.17 | 2.01 | 1.72 |
| max | 7.99 | 11.42 | 8.04 | 3.75 | 4.12 | 4.64 |
| Mean | 5.19 | 5.19 | 3.62 | 1.46 | 2.80 | 3.13 |
| STDE | 0.89 | 1.20 | 0.85 | 0.44 | 0.31 | 0.40 |
| STD | 2.34 | 3.17 | 2.24 | 1.16 | 0.82 | 1.05 |

**Table S8 continued**: General water quality indicators in canals (C) and ponds (P) for the extensive (C1/P1), semi-intensive (C2/P2) and intensive (C3/P3) aquaculture farm. Red: Not meeting Coastal Water Quality Standard for Aquaculture (CWQS Table S1). Orange: Not meeting Effluent Quality Standard for Coastal Aquaculture (FEQS Table S3).

| **Total phosphorus (mg/L) FEQS < 0.4 mg/L** | | | | | | |
| --- | --- | --- | --- | --- | --- | --- |
| Date | C1 | C2 | C3 | P1 | P2 | P3 |
| 29.3.17 | 1.026 | 1.048 | 0.506 | 0.996 | 0.445 | 0.445 |
| 21.6.17 | 0.858 | 0.827 | 0.584 | 0.768 | 0.325 | 1.380 |
| 19.7.17 | 0.757 | 0.799 | 0.772 | 0.261 | 0.184 | 0.880 |
| 15.8.17 | 0.481 | 0.640 | 0.390 | 0.270 | 0.289 | 1.010 |
| 12.9.17 | 0.735 | 0.708 | 0.790 | 0.359 | 0.286 | 0.741 |
| 12.10.17 | 0.024 | 0.014 | 0.016 | 0.012 | 0.009 | 0.010 |
| 20.12.17 | 0.160 | 0.159 | 0.215 | 0.150 | 0.157 | 0.151 |
| 12.1.18 | 0.264 | 0.264 | 0.267 | 0.268 | 0.287 | 0.286 |
| min | 0.024 | 0.014 | 0.016 | 0.012 | 0.009 | 0.010 |
| max | 1.026 | 1.048 | 0.790 | 0.996 | 0.445 | 1.380 |
| Mean | 0.538 | 0.557 | 0.442 | 0.385 | 0.248 | 0.613 |
| STDE | 0.128 | 0.130 | 0.096 | 0.116 | 0.046 | 0.166 |
| STD | 0.361 | 0.367 | 0.273 | 0.329 | 0.130 | 0.470 |
| **Ortho phosphate phosphorus (mg/L) CWQS ≤ 0.045 mg/L** | | | | | | |
| Date | C1 | C2 | C3 | P1 | P2 | P3 |
| 29.3.17 | 0.381 | 0.467 | 0.435 | 0.973 | 0.007 | 0.007 |
| 21.6.17 | 0.436 | 0.410 | 0.324 | 0.230 | 0.034 | 1.147 |
| 19.7.17 | 0.447 | 0.452 | 0.555 | 0.156 | 0.006 | 0.593 |
| 15.8.17 | 0.557 | 0.370 | 2.936 | 0.121 | 0.052 | 1.041 |
| 12.9.17 | 0.471 | 0.507 | 0.492 | 0.196 | 0.234 | 0.652 |
| 12.10.17 | 0.005 | 0.007 | 0.005 | 0.003 | 0.006 | 0.016 |
| 20.12.17 | 0.010 | 0.004 | 0.006 | 0.005 | 0.006 | 0.009 |
| 12.1.18 | 0.009 | 0.006 | 0.008 | 0.009 | 0.004 | 0.003 |
| min | 0.005 | 0.004 | 0.005 | 0.003 | 0.004 | 0.003 |
| max | 0.557 | 0.507 | 2.936 | 0.973 | 0.234 | 1.147 |
| Mean | 0.290 | 0.278 | 0.595 | 0.212 | 0.044 | 0.433 |
| STDE | 0.084 | 0.081 | 0.344 | 0.113 | 0.028 | 0.173 |
| STD | 0.238 | 0.229 | 0.973 | 0.320 | 0.079 | 0.489 |
| **Suspended solids (mg/L) FEQS < 70 mg/L** | | | | | | |
| Date | C1 | C2 | C3 | P1 | P2 | P3 |
| 29.3.17 | 38.67 | 38.33 | 29.33 | 28.67 | 100.33 | 100.33 |
| 21.6.17 | 51.00 | 74.00 | 29.00 | 23.00 | 40.50 | 15.00 |
| 19.7.17 | 29.00 | 37.50 | 21.50 | 19.50 | 39.00 | 26.50 |
| 15.8.17 | 30.50 | 32.00 | 62.10 | 23.50 | 74.33 | 19.50 |
| 12.9.17 | 44.00 | 29.50 | 27.50 | 20.50 | 25.50 | 28.00 |
| 12.10.17 | 17.00 | 18.50 | 11.50 | 19.50 | 25.00 | 68.50 |
| 20.12.17 | 39.00 | 31.50 | 100.50 | 46.50 | 32.00 | 48.00 |
| 12.1.18 | 24.00 | 33.00 | 41.00 | 32.00 | 76.00 | 91.50 |
| min | 17.00 | 18.50 | 11.50 | 19.50 | 25.00 | 15.00 |
| max | 51.00 | 74.00 | 100.50 | 46.50 | 100.33 | 100.33 |
| Mean | 34.15 | 36.79 | 40.30 | 26.65 | 51.58 | 49.67 |
| STDE | 3.92 | 5.73 | 10.08 | 3.25 | 9.95 | 11.80 |
| STD | 11.10 | 16.21 | 28.52 | 9.18 | 28.14 | 33.36 |

**Table S8 continued**: General water quality indicators in canals (C) and ponds (P) for the extensive (C1/P1), semi-intensive (C2/P2) and intensive (C3/P3) aquaculture farm. Red: Not meeting Coastal Water Quality Standard for Aquaculture (CWQS Table S1). Orange: Not meeting Effluent Quality Standard for Coastal Aquaculture (FEQS Table S3).

| **pH CWQS 7.0-8.5 FEQS 6.5-9.0** | | | | | | |
| --- | --- | --- | --- | --- | --- | --- |
| Date | C1 | C2 | C3 | P1 | P2 | P3 |
| 29.3.17 | 7.08 | 7.54 | 7.44 | 8.55 | 8.17 | 7.36 |
| 21.6.17 | 7.61 | 7.40 | 7.53 | 7.89 | 8.14 | 7.22 |
| 19.7.17 | 7.36 | 6.98 | 7.35 | 7.03 | 7.86 | 8.47 |
| 15.8.17 | 7.39 | 7.52 | 7.44 | 7.95 | 8.73 | 8.10 |
| 12.9.17 | 9.21 | 7.03 | 7.51 | 8.13 | 8.73 | 7.06 |
| 12.10.17 | 7.34 | 7.34 | 7.37 | 7.58 | 9.08 | 7.49 |
| 20.12.17 | 7.72 | 8.12 | 7.75 | 8.15 | 8.92 | 7.80 |
| 12.1.18 | 7.41 | 7.48 | 7.35 | 7.53 | 7.97 | 7.54 |
| min | 7.08 | 6.98 | 7.35 | 7.03 | 7.86 | 7.06 |
| max | 9.21 | 8.12 | 7.75 | 8.55 | 9.08 | 8.47 |
| Mean | 7.64 | 7.43 | 7.47 | 7.85 | 8.45 | 7.63 |
| STDE | 0.23 | 0.12 | 0.05 | 0.16 | 0.17 | 0.17 |
| STD | 0.66 | 0.35 | 0.13 | 0.47 | 0.47 | 0.47 |
| **Temperature (ºC)** | | | | | | |
| Date | C1 | C2 | C3 | P1 | P2 | P3 |
| 29.3.17 | 32.70 |  |  | 32.70 |  |  |
| 21.6.17 | 32.5 | 31.8 | 33.2 | 32.9 | 33.2 | 34.8 |
| 19.7.17 | 30.8 | 30.3 | 29.9 | 30.5 | 30.2 | 30.6 |
| 15.8.17 | 32.4 | 31.9 | 33.4 | 33.4 | 33.2 | 35.3 |
| 12.9.17 | 31.7 | 33.1 | 33.3 | 33.8 | 33.8 | 34.2 |
| 12.10.17 | 32.9 | 32.9 | 31.8 | 31.9 | 32.2 | 32.1 |
| 20.12.17 | 25.5 | 25.7 | 24.5 | 22.9 | 25.1 | 25.8 |
| 12.1.18 | 26.4 | 25.1 | 26.4 | 25.9 | 24.5 | 26.6 |
| min | 25.50 | 25.10 | 24.50 | 22.90 | 24.50 | 25.80 |
| max | 32.90 | 33.10 | 33.40 | 33.80 | 33.80 | 35.30 |
| Mean | 30.61 | 30.11 | 30.36 | 30.50 | 30.31 | 31.34 |
| STDE | 1.05 | 1.27 | 1.37 | 1.41 | 1.49 | 1.46 |
| STD | 2.96 | 3.35 | 3.61 | 3.98 | 3.94 | 3.88 |
| **Electric conductivity (mS/cm)** | | | | | | |
| Date | C1 | C2 | C3 | P1 | P2 | P3 |
| 29.3.17 | 1.53 | 2.91 | 10.92 | 25.01 | 14.47 | 8.81 |
| 21.6.17 | 1.96 | 2.10 | 5.60 | 7.96 | 12.23 | 7.21 |
| 19.7.17 | 1.117 | 1.247 | 1.627 | 6.630 | 0.106 | 5.640 |
| 15.8.17 | 1.060 | 4.280 | 0.970 | 7.380 | 10.720 | 6.230 |
| 12.9.17 | 1.918 | 1.180 | 2.280 | 3.620 | 6.750 | 5.750 |
| 12.10.17 | 1.495 | 1.167 | 2.211 | 1.464 | 5.330 | 4.930 |
| 20.12.17 | 0.815 | 1.482 | 6.840 | 1.501 | 4.870 | 4.080 |
| 12.1.18 | 1.046 | 1.473 | 4.530 | 1.737 | 5.290 | 5.350 |
| min | 0.82 | 1.17 | 0.97 | 1.46 | 0.11 | 4.08 |
| max | 1.96 | 4.28 | 10.92 | 25.01 | 14.47 | 8.81 |
| Mean | 1.37 | 1.98 | 4.37 | 6.91 | 7.47 | 6.00 |
| STDE | 0.15 | 0.39 | 1.19 | 2.76 | 1.65 | 0.52 |
| STD | 0.42 | 1.10 | 3.35 | 7.80 | 4.68 | 1.46 |

**Table S8 continued**: General water quality indicators in canals (C) and ponds (P) for the extensive (C1/P1), semi-intensive (C2/P2) and intensive (C3/P3) aquaculture farm. Red: Not meeting Coastal Water Quality Standard for Aquaculture (CWQS Table S1). Orange: Not meeting Effluent Quality Standard for Coastal Aquaculture (FEQS Table S3).

| **Faecal coliforms (#/100 mL) CWQS ≤ 70** | | | | | | |
| --- | --- | --- | --- | --- | --- | --- |
| Date | C1 | C2 | C3 | P1 | P2 | P3 |
| 06.2017 | 307500 | 295000 | 115000 | 34500 | 35500 | 350000 |
| 07.2017 | 830000 | 800000 | 225000 | 10000 | 102500 | 75000 |
| 08.2017 | 237500 | 140000 | 160000 | 165000 | 45000 | 45000 |
| 12.2017 | 310000 | 18750 | 42750 | 13000 | 10000 | 14300 |
| 01.2018 | 450000 | 52500 | 42500 | 23000 | 12250 | 40000 |
| 02.2018 | 183600 | 35000 | 22500 | 8500 | 1000 | 10000 |
| min | 183600 | 18750 | 22500 | 8500 | 1000 | 10000 |
| max | 830000 | 800000 | 225000 | 165000 | 102500 | 350000 |
| Mean | 386433 | 223542 | 101292 | 42333 | 34375 | 89050 |
| STDE | 95949 | 122629 | 32675 | 24855 | 15224 | 53064 |
| STD | 235026 | 300377 | 80038 | 60882 | 37290 | 129981 |

**Table S9**: Micronutrients and metals in filtered water samples. P = Pond, C= Canal. Red: Not meeting Coastal Water Quality Standard for Aquaculture (CWQS Table S1).

| Parameter | P1  April 2017  June 2018 | P2  April 2017  June 2018 | P3  April 2017  June 2018 | C1  April 2017  June 2018 | C2  April 2017  June 2018 | C3  April 2017  June 2018 |
| --- | --- | --- | --- | --- | --- | --- |
| Ca (mg/L) | 79.4  12.7 | 72.8  28.2 | 87.3  35.1 | 40.4  40.6 | 45.0  41.6 | 78.7  43.7 |
| Mg (mg/L) | 342  52.6 | 333  136 | 208  158 | 28.2  31.3 | 52.3  42.0 | 241  114 |
| Na (mg/L) | 2389  358 | 2387  1131 | 1402  1278 | 179  227 | 344  262 | 1719  956 |
| K (mg/L) | 92.5  29.9 | 102  64.3 | 57.7  43.4 | 16.1  19.4 | 27.3  17.4 | 74.6  51.6 |
| Ba (mg/L) | 0.063  0.010 | 0.033  0.010 | 0.014  0.012 | 0.038  0.035 | 0.037  0.034 | 0.039  0.019 |
| Fe (mg/L) | <0.01  0.013 | <0.01  0.007 | <0.01  0.008 | 0.032  0.027 | 0.026  0.030 | <0.01  0.008 |
| Mn (mg/L) | 0.105  0.003 | 0.004  0.006 | 0.023  0.125 | 0.231  0.135 | 0.247  0.168 | 0.342  0.004 |
| Al (mg/L) | 0.023  0.010 | 0.023  0.008 | 0.011  0.010 | 0.020  0.016 | 0.022  0.016 | 0.023  0.009 |
| Zn (mg/L) | <0.01  0.002 | <0.01  0.003 | <0.01  0.009 | <0.01  0.010 | <0.01  0.010 | <0.01  0.004 |
| Pb (mg/L) | <0.005  <0.005 | <0.005  <0.005 | <0.005  <0.005 | <0.005  <0.005 | <0.005  <0.005 | <0.005  <0.005 |
| Cu (mg/L) | 0.010  0.001 | 0.063  0.001 | 0.077  0.004 | 0.002  0.003 | 0.002  0.002 | 0.007  0.001 |
| As (mg/L) | 0.008  0.005 | 0.008  0.007 | 0.008  0.008 | <0.005  <0.005 | <0.005  <0.005 | <0.005  0.006 |
| Cd (mg/L) | <0.001  <0.001 | <0.001  <0.001 | <0.001  <0.001 | <0.001  <0.001 | <0.001  <0.001 | <0.001  <0.001 |

**Table S9 continued**: Micronutrients and metals in filtered water samples. P = Pond, C= Canal. Red: Not meeting Coastal Water Quality Standard for Aquaculture (CWQS Table S1).

| Parameter | P1  April 2017  June 2018 | P2  April 2017  June 2018 | P3  April 2017  June 2018 | C1  April 2017  June 2018 | C2  April 2017  June 2018 | C3  April 2017  June 2018 |
| --- | --- | --- | --- | --- | --- | --- |
| Sb (mg/L) | <0.005  <0.005 | <0.005  <0.005 | <0.005  <0.005 | <0.005  <0.005 | <0.005  <0.005 | <0.005  <0.005 |
| Si (mg/L) | 6.84  3.3 | 10.1  13.1 | 6.55  0.9 | 5.61  6.3 | 5.51  6.4 | 7.46  4.1 |
| Ni (mg/L) | 0.003  0.004 | 0.003  0.003 | 0.006  0.005 | 0.009  0.007 | 0.008  0.007 | 0.002  0.002 |
| Cr (mg/L) | <0.001  <0.001 | <0.001  <0.001 | <0.001  <0.001 | <0.001  <0.001 | <0.001  <0.001 | <0.001  <0.001 |
| Sr (mg/L) | 1.860  0.209 | 0.950  0.534 | 0.909  0.621 | 0.191  0.224 | 0.308  0.247 | 1.074  0.580 |

**Table S10**: Environmental risk assessment of antibiotics and herbicides/pesticides in surface water.

| Substance | Species^1^ | Study details | Toxicity endpoint – water (µg/L)^2^ | Maximum concentration observed in water (µg/L)^3^ | Toxicity Exposure Ratio (TER) | Risk quotient^4^ | Source of toxicity data^5^ |
| --- | --- | --- | --- | --- | --- | --- | --- |
| Tetracycline | *Lemna gibba* | 7-day OECD TG 221 | E_b_C_50_ = 1000 | 0.238 | 4201.68 | 0.02 | ^11^ |
| Endrofloxacine | *Lemna gibba* | 7-day OECD TG 221 | E_b_C_50_ > 84.0 | 0.154 | 545.45 | 0.02 | ^11^ |
| Diuron | *Lemna gibba* | 7-day OECD TG 221 | E_r_C_50_ = 18.3 | 0.278 | 65.83 | 0.15 | ^12^ |
| Ethoprophos | *Daphnia magna* | 21-day OECD TG 211 | NOEC = 2.0 | 0.015 | 133.33 | 0.08 | ^13^ |
| Atrazine | *Lemna gibba* | 7-day OECD TG 221 | E_r_C_50_ = 37.0 | 0.111 | 333.33 | 0.03 | ^14^ |

^1^ Data is available on a number of standard test species, however, only the results for the species most sensitive to that substance are provided here as this is the worst-case scenario (i.e. if this species passes the risk assessment then all other species with higher toxicity endpoints would also pass the risk assessment) ^2^ E_b_C_50_ is the concentration where a 50% effect on biomass was observed; E_r_C_50_ is the concentration where a 50% effect on growth rate was observed; NOEC is the No Observed Effect Concentration (the highest concentration in the toxicity test that did not show statistically significant differences in endpoints from the control) ^3^ The maximum value from all replicates from all three canals and ponds was selected as a worst-case scenario for the risk assessment. 4 The risk quotient value is calculated using the equation $RQ=\frac{Trigger}{TER}$. The trigger for all substances in this table was 10 (based on the standard trigger values used in the risk assessment of agricultural chemicals in the EU). ^5^ VSDB is the Veterinary Substance Database, University of Hertfordshire; EFSA is the European Food Safety Authority; US EPA is the United States Environmental Protection Agency

**Table S11**: Comparison of the observed maximum residues in shrimp and their accepted maximum residue levels for five different micropollutants. All observed values were well below the MRLs for all substances.

| Substance | Maximum concentration in shrimp | | MRL (mg/kg) ^1^ | MRL reference ^2^ |
| --- | --- | --- | --- | --- |
|  | ng/g | mg/kg |  |  |
| Tetracycline | 4.55 | 0.00455 | 0.01 | EU commission No. 37/2010 |
| Endrofloxacine | 3.80 | 0.00380 | 0.01 | EU commission No. 37/2010 |
| Diuron | 3.64 | 0.00364 | 0.05 | EU pesticides database |
| Ethoprophos | 0.91 | 0.00091 | 0.01 | EU pesticides database |
| Atrazine | 2.82 | 0.00282 | 0.05 | EU pesticides database |

1 Maximum residue levels (MRL) can differ for different products. In cases where no specific MRLs are available for shrimp, then the lowest MRL value associated with aquaculture products or animal products for that substance was used.

^2^ EU pesticides database: http://ec.europa.eu/food/plant/pesticides/eu-pesticides-database/public/?event=pesticide.residue.selection&language=EN

**Figure S1**: a) Cluster analysis, and b) relative abundance of taxa at rank phylum for 16s rRNA gene sequencing reads. P = Pond, C= Canal, for the extensive (C1/P1), semi-intensive (C2/P2) and intensive (C3/P3) aquaculture farm. Replicates are indicated as a/b.


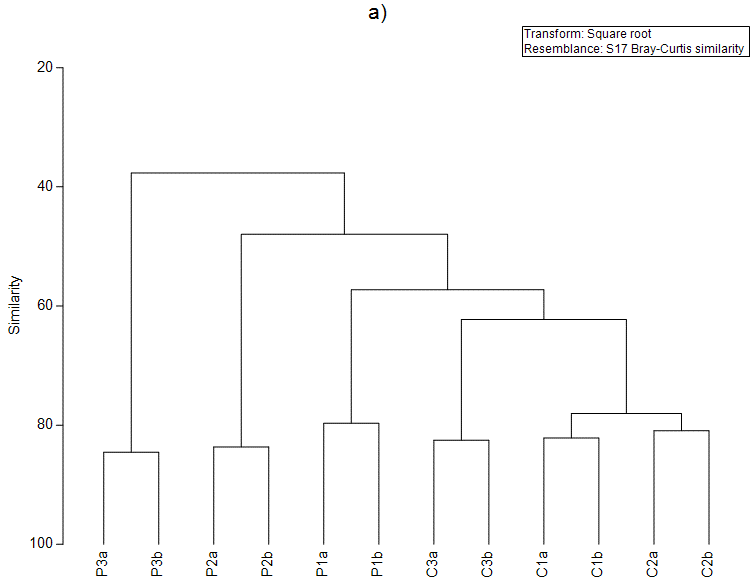


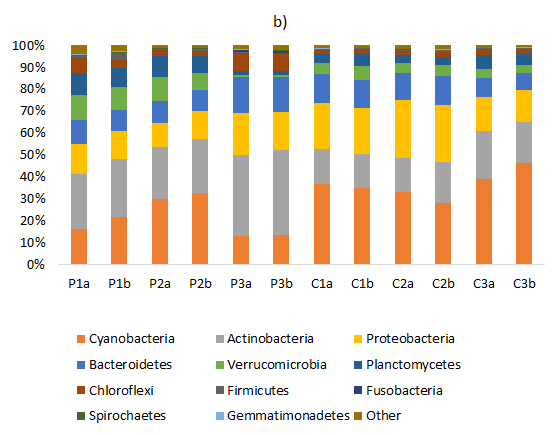


**REFERENCES**

1. Kozich, J. J.; Westcott, S. L.; Baxter, N. T.; Highlander, S. K.; Schloss, P. D., Development of a Dual-Index Sequencing Strategy and Curation Pipeline for Analyzing Amplicon Sequence Data on the MiSeq Illumina Sequencing Platform. *Applied and Environmental Microbiology* **2013,** *79*, (17), 5112.

2. Caporaso, J. G.; Kuczynski, J.; Stombaugh, J.; Bittinger, K.; Bushman, F. D.; Costello, E. K.; Fierer, N.; Peña, A. G.; Goodrich, J. K.; Gordon, J. I.; Huttley, G. A.; Kelley, S. T.; Knights, D.; Koenig, J. E.; Ley, R. E.; Lozupone, C. A.; McDonald, D.; Muegge, B. D.; Pirrung, M.; Reeder, J.; Sevinsky, J. R.; Turnbaugh, P. J.; Walters, W. A.; Widmann, J.; Yatsunenko, T.; Zaneveld, J.; Knight, R., QIIME allows analysis of high-throughput community sequencing data. *Nature Methods* **2010,** *7*, 335.

3. Edgar, R. C., Search and clustering orders of magnitude faster than BLAST. *Bioinformatics* **2010,** *26*, (19), 2460-2461.

4. Wang, Q.; Garrity, G. M.; Tiedje, J. M.; Cole, J. R., Naïve Bayesian Classifier for Rapid Assignment of rRNA Sequences into the New Bacterial Taxonomy. *Applied and Environmental Microbiology* **2007,** *73*, (16), 5261-5267.

5. DeSantis, T. Z.; Hugenholtz, P.; Larsen, N.; Rojas, M.; Brodie, E. L.; Keller, K.; Huber, T.; Dalevi, D.; Hu, P.; Andersen, G. L., Greengenes, a Chimera-Checked 16S rRNA Gene Database and Workbench Compatible with ARB. *Applied and Environmental Microbiology* **2006,** *72*, (7), 5069-5072.

6. Bai, Y.; Meng, W.; Xu, J.; Zhang, Y.; Guo, C., Occurrence, distribution and bioaccumulation of antibiotics in the Liao River Basin in China. *Environmental Science: Processes & Impacts* **2014,** *16*, (3), 586-593.

7. Hale, S. E.; Martin, T. J.; Goss, K.-U.; Arp, H. P. H.; Werner, D., Partitioning of organochlorine pesticides from water to polyethylene passive samplers. *Environmental Pollution* **2010,** *158*, (7), 2511-2517.

8. Gomi, R.; Matsuda, T.; Matsui, Y.; Yoneda, M., Fecal source tracking in water by next-generation sequencing technologies using host-specific Escherichia coli genetic markers. *Environmental science & technology* **2014,** *48*, (16), 9616-9623.

9. Harms, G.; Layton, A. C.; Dionisi, H. M.; Gregory, I. R.; Garrett, V. M.; Hawkins, S. A.; Robinson, K. G.; Sayler, G. S., Real-time PCR quantification of nitrifying bacteria in a municipal wastewater treatment plant. *Environmental science & technology* **2003,** *37*, (2), 343-351.

10. Szekeres, E.; Chiriac, C. M.; Baricz, A.; Szőke-Nagy, T.; Lung, I.; Soran, M.-L.; Rudi, K.; Dragos, N.; Coman, C., Investigating antibiotics, antibiotic resistance genes, and microbial contaminants in groundwater in relation to the proximity of urban areas. *Environmental Pollution* **2018,** *236*, 734-744.

11. VSDB Veterinary Substance Database. <https://sitem.herts.ac.uk/aeru/vsdb/>

12. EFSA *Conclusion regarding the peer review of the pesticide risk assessment of the active substance diuron.*; EFSA Scientific Report 25; European Food Safety Authority: 2005; pp 1-58.

13. EFSA *Conclusion regarding the peer review of the pesticide risk assessment of the active substance ethoprophos*; EFSA Scientific Report 66; European Food Safety Authority: 2006; pp 1-72.

14. EPA, U. *Decision documents for atrazine. IRED.* ; Office of prevention, pesticides and toxic substances: Washington., 2006.
